# Evolutionary relationships and expression analysis of EUL domain proteins in rice (*Oryza sativa*)

**DOI:** 10.1186/s12284-017-0164-3

**Published:** 2017-05-30

**Authors:** Kristof De Schutter, Mariya Tsaneva, Shubhada R. Kulkarni, Pierre Rougé, Klaas Vandepoele, Els J. M. Van Damme

**Affiliations:** 1 Laboratory Biochemistry and Glycobiology, Department of Molecular Biotechnology, Ghent University, Coupure links 653, B-9000 Ghent, Belgium; 2 Department of Plant Biotechnology and Bioinformatics, Ghent University, Technologiepark 927, B-9052 Ghent, Belgium; 3 VIB Center for Plant Systems Biology, Technologiepark 927, B-9052 Ghent, Belgium; 4 Bioinformatics Institute Ghent, Ghent University, Technologiepark 927, B-9052 Ghent, Belgium; 5 UMR 152 PHARMA-DEV, Université de Toulouse, IRD, UPS, Chemin des Maraîchers 35, 31400 Toulouse, France

**Keywords:** EUL, Lectin, Carbohydrate, Rice, Domain architecture, Phylogeny

## Abstract

**Background:**

Lectins, defined as ‘Proteins that can recognize and bind specific carbohydrate structures’, are widespread among all kingdoms of life and play an important role in various biological processes in the cell. Most plant lectins are involved in stress signaling and/or defense. The family of *Euonymus*-related lectins (EULs) represents a group of stress-related lectins composed of one or two EUL domains. The latter protein domain is unique in that it is ubiquitous in land plants, suggesting an important role for these proteins.

**Results:**

Despite the availability of multiple completely sequenced rice genomes, little is known on the occurrence of lectins in rice. We identified 329 putative lectin genes in the genome of *Oryza sativa* subsp. japonica belonging to nine out of 12 plant lectin families. In this paper, an in-depth molecular characterization of the EUL family of rice was performed. In addition, analyses of the promoter sequences and investigation of the transcript levels for these EUL genes enabled retrieval of important information related to the function and stress responsiveness of these lectins. Finally, a comparative analysis between rice cultivars and several monocot and dicot species revealed a high degree of sequence conservation within the EUL domain as well as in the domain organization of these lectins.

**Conclusions:**

The presence of EULs throughout the plant kingdom and the high degree of sequence conservation in the EUL domain suggest that these proteins serve an important function in the plant cell. Analysis of the promoter region of the rice EUL genes revealed a diversity of stress responsive elements. Furthermore analysis of the expression profiles of the EUL genes confirmed that they are differentially regulated in response to several types of stress. These data suggest a potential role for the EULs in plant stress signaling and defense.

**Electronic supplementary material:**

The online version of this article (doi:10.1186/s12284-017-0164-3) contains supplementary material, which is available to authorized users.

## Background

Proteins of non-immune origin with at least one non-catalytic domain that can recognize and reversibly bind to specific carbohydrate structures are referred to as ‘lectins’. The occurrence of carbohydrate-binding proteins in all kingdoms of life illustrates their importance. Indeed, the specific interaction between lectins and their corresponding carbohydrate partner(s), either occurring as a free ligand or as part of a glycoconjugate, mediates a multitude of biological processes. These interactions can relay cellular signaling and are of utmost importance for defense reactions, stress signaling, growth and development.

In plants, lectins are divided into 12 families of structurally and evolutionary related proteins based on the presence of a conserved carbohydrate-recognition domain (Van Damme et al. 2008). Like animals, plants can experience several forms of stress due to e.g. environmental conditions or biological agents. Because of their sessile lifestyle, plants cannot move away from these stresses, and therefore have developed a sophisticated system to recognize the different stressors and initiate a specific response to this stress. It was shown that carbohydrate-binding proteins play a pivotal role in plant defense (review De Schutter and Van Damme 2015).

In addition to the structural classification, plant lectins can also be subdivided in 2 classes depending on their expression pattern. The first class groups all lectins that are constitutively expressed. These lectins are usually present at high concentrations in specific cells and organs (e.g. seeds and specialized vegetative tissues), suggesting a dual role as a storage protein involved in plant defense. The second class groups all lectins which are present at a low basal level but when plants are exposed to biotic or abiotic stresses, the expression of these lectins is significantly upregulated. At present, the expression of at least 6 different lectin domains from plants has been shown to be stress regulated. Most of these stress related lectins locate to the cytoplasm and the nucleus of plant cells (Lannoo and Van Damme 2010).

The EUL family, grouping all proteins that show homology to the *Euonymus europeaus* agglutinin (EEA) (Petryniak et al. 1977; Fouquaert et al. 2008), belongs to the group of stress related lectins. In contrast to many other lectin families that occur only in some plant families, the EUL family represents a group of nucleocytoplasmic proteins that are found throughout the plant kingdom, suggesting they fulfill an essential role in plants (Fouquaert et al. 2009). The genome of *Arabidopsis thaliana* harbours only one *EUL* gene, referred to as *ArathEULS3*. The expression of this gene is upregulated after exposure to some plant hormones, drought and salt stress as well as *Pseudomonas syringae* infection (Li et al. 2014; Van Hove et al. 2014). Furthermore, it was shown that changes in expression levels of the lectin are accompanied with altered levels of stress resistance. Overexpression of *ArathEULS3* enhanced drought resistance (Li et al. 2014) and plants showed less disease symptoms after *P. syringae* infection compared to wild type plants or plants with lower transcript levels of the lectin gene (Van Hove et al. 2015).

*Oryza sativa* L., or Asian cultivated rice, is one of the most important food crops and staple foods. More than half of the world population is dependent on rice, it has shaped their diet, culture and economics. Rice breeding and cultivation has given rise to the existence of several subspecies. The sticky, short-grained japonica rice is grown in dry fields at higher altitudes in temperate environments, whereas the non-sticky, long-grained indica rice grows mostly submerged in lowlands in tropical and subtropical environments. Besides these morphological and agronomical differences, both rice varieties show distinct physiological and biochemical features. Amongst other, these features translate in differences in stress resistance (Yang et al. 2014; Hu et al. 2014; Liu et al. 2010). Although the phenotypic differences between the two subspecies are well studied, the molecular mechanisms behind these traits are only poorly characterized. Since plant lectins play an important role in plant defense and stress signaling, we focus on the identification of lectins in rice.

In this paper we identified the putative lectin genes encoded in the japonica rice genome. An in-depth molecular characterization and analysis of the transcription profiles of the EUL family was performed under abiotic stress conditions. The strong conservation of the EUL lectin domain and the stress regulated transcription profiles suggest that this family of lectins fulfills an important role in plant stress signaling.

## Results

### Distribution and organization of lectin sequences in the *O. sativa* genome

Lectin genes in the genome of *O. sativa* subsp. japonica were identified through extensive BLAST searches. Using the protein sequences for each of the representative members for the different plant lectin families, pFam identifiers and conserved domains, a total of 329 putative lectin sequences were identified in the japonica genome (Table 1). All these lectin sequences were further classified into nine of the 12 plant lectin families (Table 1). No homologs representing lectins belonging to the *Agaricus bisporus* agglutinin, Amaranthin and Cyanovirin families were found. A large variation exists in the abundance of lectin genes for the different lectin families. The *Galanthus nivalis* agglutinin (GNA) family represents the largest lectin family with 134 genes (40.7%) whereas the *Robinia pseudoacacia* chitinase-related agglutinin (CRA) family is the smallest with only 2 genes (0.6%). Lectin sequences of the GNA and legume lectin families account for 72.3% of all lectin sequences in rice.

**Table 1:** Predicted lectin genes in the *Oryza sativa* genome

Lectin domain	Model lectin	*O. sativa* japonica	*O. sativa* indica
ABA domain	*Agaricus bisporus* agglutinin	0	0
Amaranthin domain	*Amaranthus caudatus* agglutinin	0	0
CRA domain	*Robinia pseudoacacia* chitinase-related agglutinin	2	4
Cyanovirin domain	*Nostoc ellipsosporum* agglutinin	0	0
EUL domain	*Euonymus europaeus* agglutinin	5	5
GNA domain	*Galanthus nivalis* agglutinin	134	94
Hevein domain	*Hevea brasiliensis* agglutinin	10	10
JRL domain	*Artocarus integer* agglutinin	30	33
Legume domain	*Glycine max* agglutinin	104	84
LysM domain	*Brassica juncea* LysM domain	20	22
Nictaba domain	*Nicotiana tabacum* agglutinin	20	22
Ricin-B domain	*Ricinus communis* agglutinin	4	4

A comparative analysis between the subspecies japonica and indica revealed only minor differences in the number of lectin sequences for most lectin families, but considerably more lectin homologs were retrieved from the japonica genome for the legume family and the GNA family (Table 1). This study will focus on the lectin sequences retrieved from the genome sequence *O. sativa* subsp. japonica.

### The EUL family in the *O. sativa* genome

**Fig. 1:**
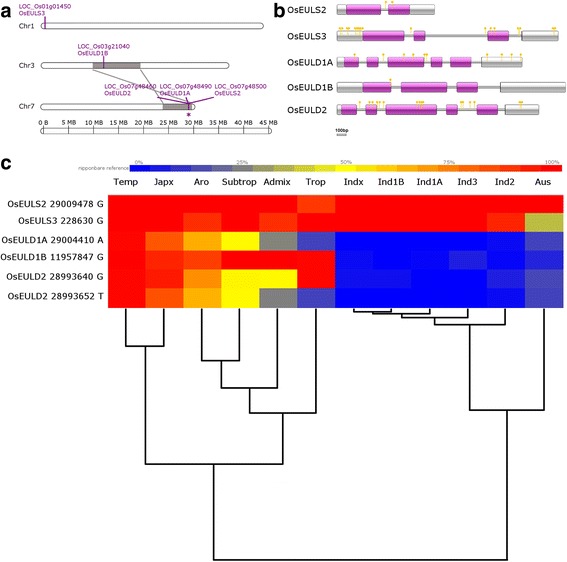
Comparison of DNA sequences encoding EUL homologs from *O. sativa*. **a**. Distribution and duplication of EUL genes in the *Oryza sativa* subsp. japonica genome. Sequences for the different EULs were mapped on the chromosomes based on their start position and annotated with their locus number. Blocks containing EUL genes resulting from segmental duplication events are indicated in gray and connected by lines. Tandem duplications are indicated by an asterisk. The chromosome map was prepared using MapChart and drawn to scale. **b**. Intron-exon structure of the EUL genes. Shown in white are the 5′ and 3′ UTRs, exons are shown in purple. Introns are represented by grey bars between the exons. SNPs identified by SNP-seek are indicated in yellow.**c**. Distribution of SNP alleles in different rice varieties. The percentage of the Nipponbare reference allele in the different subvarieties is indicated by the heat map. The dendrogram is generated by DendroUPGMA (http://genomes.urv.cat/UPGMA/) (Garcia-Vallve et al. 1999)

In contrast to the Arabidopsis genome which contains a single gene belonging to the EUL family, five EUL genes were identified in the genomes of rice (Table 1), further referred to OsEULS2, OsEULS3, OsEULD1A, OsEULD1B and OsEULD2 according to the nomenclature suggested by Fouquaert et al. (2009). The S-type lectins are composed of a single EUL domain, whereas the D-type lectins contain two tandem arranged EUL domains. These five EUL genes are distributed over three chromosomes, in particular chromosomes 1, 3 and 7 (Fig. 1a). When mapping segmental duplications, a duplication block was identified linking LOC_Os03g21040 (chromosome 3) with LOC_Os07g48460 (chromosome 7). Further analysis of tandem duplicated genes revealed a duplication block containing the three EUL genes on chromosome 7 (Fig. 1a).

### Genomic sequences of the EULs

Detailed analysis of the genomic sequences and the deduced coding sequences revealed that the EUL sequences contain multiple intron sequences (Fig. 1b). While the OsEULS2 gene is interrupted by a single intron, the genes encoding OsEULD1A and OsEULD2 both contain five introns, and the OsEULS3 and OsEULD1B genes contain three introns. A summary of the size for each of the different introns and exons is shown in Additional file 1: Table S1.

Several exons showed the same size (Additional file 1: Table S1) and had similar sequences at their boundaries. When plotting the exon structure of the EUL domain to the EUL protein sequence, it was clear that all EUL genes possess an exon-intron-exon transition at identical positions in the protein sequence (Additional file 2: Figure S1). Further comparison of the intron-exon structures for the different OsEUL genes revealed several similarities. Despite small differences in the size of the introns and two exons (OsEULD2 has an extended N-terminal region (nine nucleotides) and an extended linker region (162 nucleotides) compared to OsEULD1A), the intron-exon structure of the genes OsEULD2 and OsEULD1A is very similar (Fig. 1b). The exon-intron transitions of the different exons in OsEULD1A and OsEULD2 plot at the same sites of the protein (Additional file 2: Figure S1). In addition, the structure of the OsEULS3 gene correlates with that of the second domain in the OsEULD1A/D2 gene. Furthermore, when comparing the structure of OsEULD2/D1A with that of OsEULD1B, it is observed that exons 1 and 2 and exons 3 and 4 from OsEULD2/D1A are fused in OsEULD1B (Fig. 1b). Although the structure of the OsEULS2 gene deviates from the overall structure of the OsEUL genes at the 3′ end (i.e. no split stop codon), there is some similarity with the intron-exon structure of OsEULD1B showing boundaries at the same sites (Fig. 1b). This similarity in the intron-exon structures suggests a strong conservation and phylogenetic relationship between the different EUL genes from rice.

### Genomic variation in the *O. sativa* EULs

When comparing the coding sequences of the EUL genes between the japonica and indica subvarieties, it was observed that the japonica sequence coding for the N-terminal region of OsEULS3 contained an additional stretch of 33 nucleotides that was absent in the indica sequence. This sequence, located in the first half of the first exon, is flanked by sequences conserved between the two varieties. Further analysis of the coding sequences revealed only small differences between the subvarieties. Except for OsEULS2, eight SNPs were found in the coding sequences of the OsEULs. Three of these SNPs give rise to an amino acid change in the protein sequence between japonica and indica. One SNP is located in the N-terminal region of the coding sequence of OsEULD1A, and two SNPs are found in the linker sequence connecting both EUL domains of the OsEULD2 coding sequence. With the availability of SNP data from the 3000 rice genome project (SNP-seek database, IRRI), an extensive search for SNPs in the EUL genes was performed. This database contains 3024 rice genomes classified into 12 subvarieties. Japonica species are subdivided into tropical japonica (Trop), subtropical japonica (Subtrop), temperate japonica (Temp) and Japx which contains all other japonica varieties. Indica species are divided into Ind1A, Ind1B, Ind2, Ind3 and Indx which contains the remaining indica varieties. Further classes consist of aux and aromatic (Aro) rice and the admix group containing all other unassigned varieties. In total 46 SNPs were identified, of which 13 are located in exons (Fig. 1b). Of the latter SNPs, seven yielded a silent mutation and 6 SNPs introduced an amino acid change (Additional file 3: Table S2). For the SNPs that gave rise to an amino acid change, the distribution of the two alleles in the different rice subvarieties was analyzed (Fig. 1c). Almost all genomes contain the Nipponbare reference nucleotide for OsEULS2, only in 11% of the tropical japonica genomes the alternative allele is present. Similarly for OsEULS3, the Nipponbare reference allele is used preferentially, except for a low percentage of the other allele in the indica2 (7%), Aro (9%) and admix (9%) groups. However, within the Aux subvarieties only 37% has the reference allele. When analyzing the SNPs in the two-domain EULs, a clear separation is observed in the use of the alleles for the japonica and indica species (Fig. 1c). While japonica subvarieties and the aromatic and admix groups preferentially use the Nipponbare reference allele, the indica subvarieties together with the aus subvariety have a preference for the alternative allele. Tropical japonica is the exception, with a preference for the indica allele in the SNP in OsEULD1A and OsEULD2 (Fig. 1c).

### Domain architecture and structure of the rice EULs

**Fig. 2:**
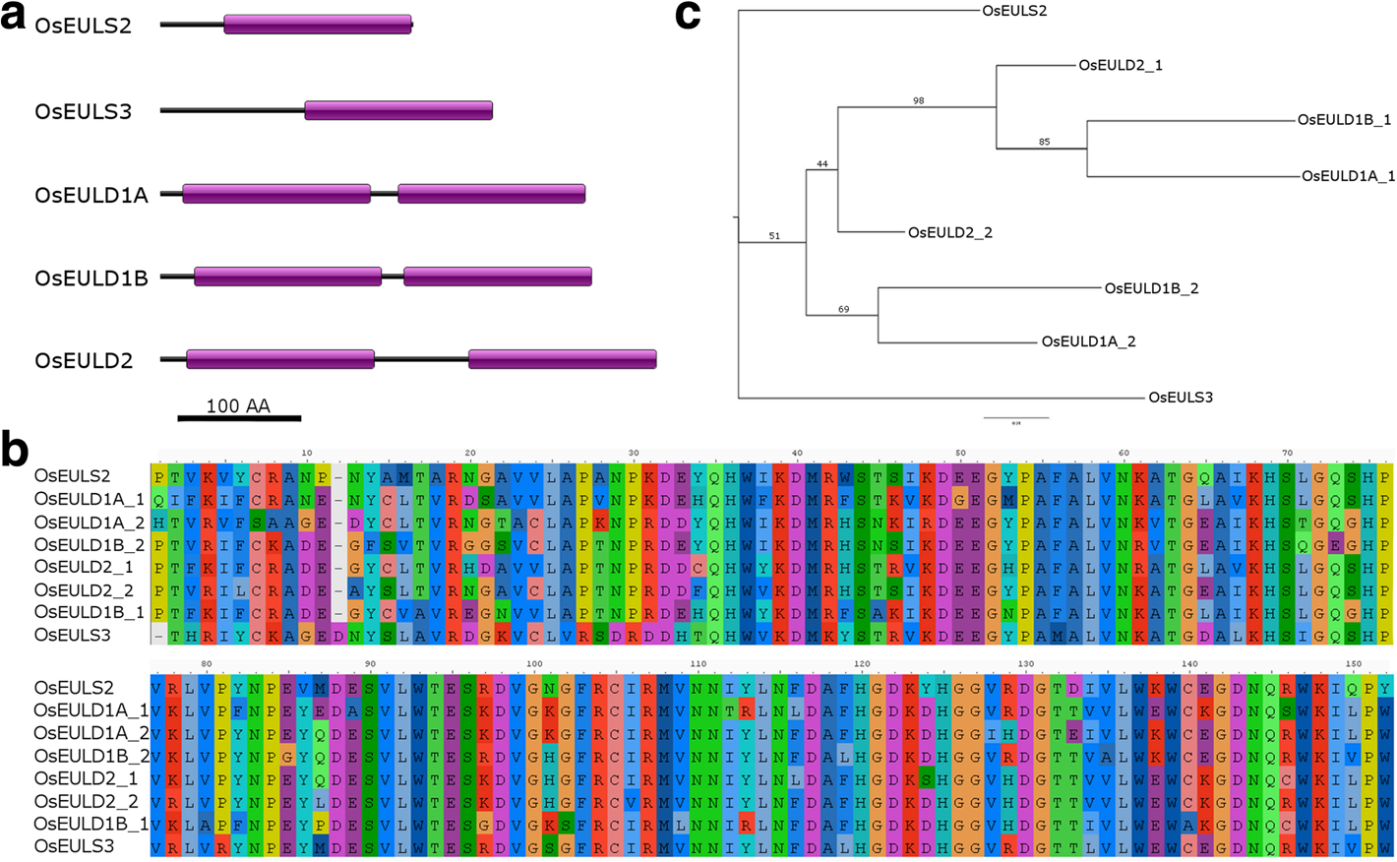
Sequence comparison of EUL homologs from *O. sativa* at protein level. **a**. Domain organization of the rice EULs. The EUL domains are represented by a purple bar. **b**. Sequence alignment of the different EUL domains. **c**. Phylogenetic relationship of EUL domains. A phylogenetic tree was generated by RaxML based on the sequence alignment shown in panel (**b**). Branch labels show bootstrapping values

Analysis of the domain structure for the different EUL lectin sequences revealed two putative lectins composed of a single EUL domain (further referred to as S-type EULs) and three proteins consisting of two tandem arrayed EUL domains (further referred to as D-type EULs) (Fig. 2a). When analyzing the sequence similarity between the different EUL domains (151 amino acids), a high degree of conservation was observed. Sequence identity between the different EUL domains can be as high as 85%, while sequence similarity reaches up to 92% (Fig. 2b, Additional file 4: Table S3). The N-terminal region preceding the EUL domain (ranging from 19 to 117 amino acids) and the linker region connecting the two EUL domains in D-type EULs (ranging from 18 to 76 amino acids) were analyzed using Interproscan and BLAST searches against the NCBI database. None of the sequences corresponded to any known protein domain. Further analysis of the protein sequences for the presence of signal peptides and transmembrane domains did not retrieve any of these sequences in the OsEULs.

Analysis of the phylogenetic relationships between the different EUL domains revealed a clustering between the different EUL domains of the D-type EULs. For this analysis the EUL domains composing the D-type lectins were separated and referred to as domain 1 and 2 for the N-terminal and C-terminal domain, respectively. Analysis of the relationship between the S-type EUL domains and the N- and C-terminal domains of the D-type EULs revealed that the C-terminal EUL domain sequences of the D-type EULs (domain 2) are more closely related to the sequences of the S-type EULs than the N-terminal EUL domain sequences of the D-type EULs (domain 1) are related to the S-type domains (Fig. 2c). This is in accordance with the higher sequence similarity between the S-type EUL domains and the C-terminal EUL domain of the D-type EULs (Additional file 4: Table S3). Within the cluster of the D-type EUL domains, the second domain of OsEULD2 is positioned between the branch containing the sequences for the N-terminal domain (domain 1) and the branch representing the C-terminal domains (domain 2) of OsEULD1A and D1B. This is also reflected in the sequence similarity between the different sequences in rice: OsEULD1A_1 and OsEULD1B_1 share the highest similarity to OsEULD2_1 (89 and 88% respectively) and the latter domain shares the highest similarity to OsEULD2_2 (90%). OsEULD1A_2 and OsEULD1B_2 share the highest similarity to OsEULD2_2 (88 and 92% respectively) (Additional file 4: Table S3).

### Molecular modeling of OsEULs

**Fig. 3:**
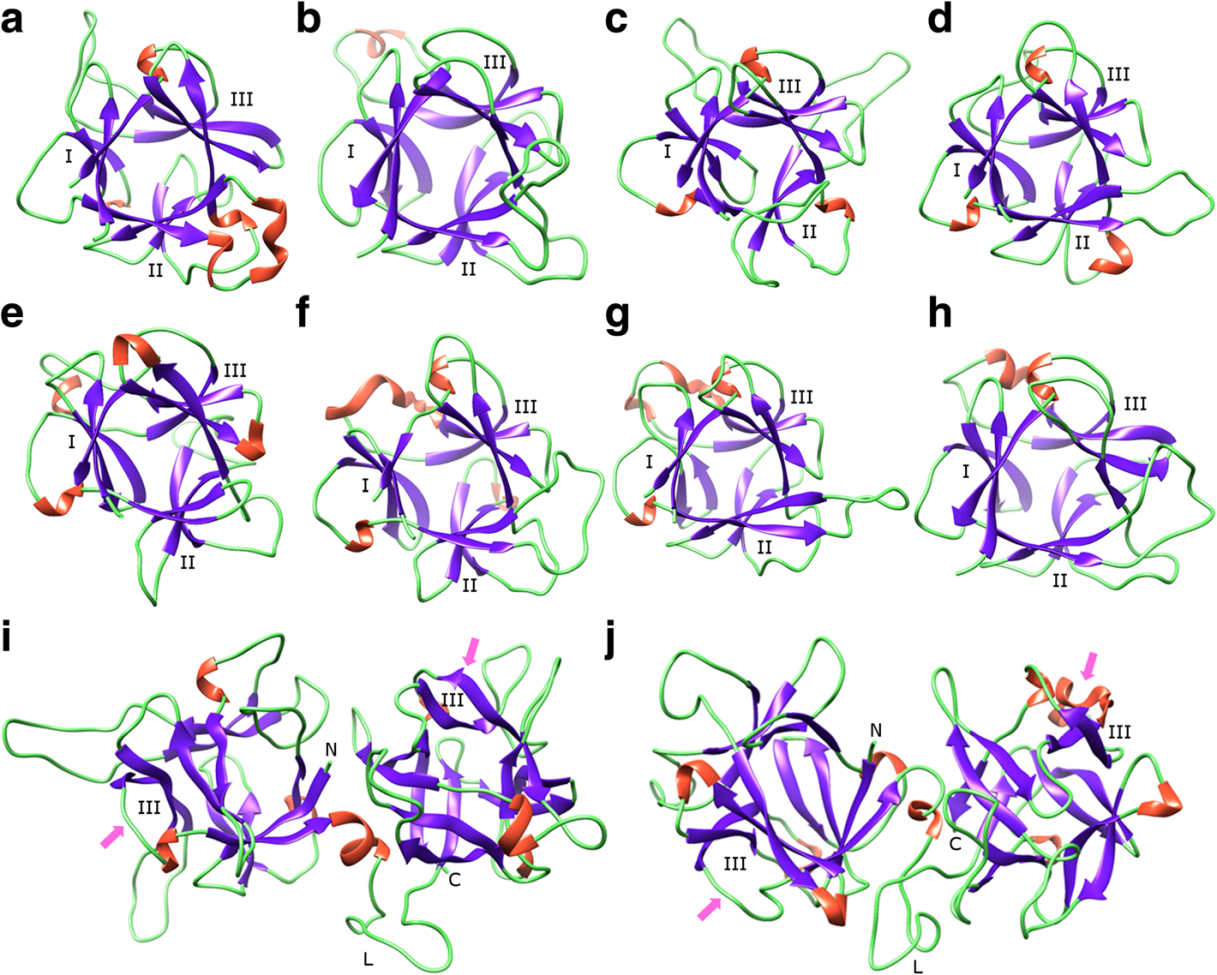
Ribbon diagrams of the β-trefoil lectin domains of *O. sativa* EULs. **a**-**h**. Ribbon diagrams of the separate EUL domains of OsEULS2 (**a**), OsEULS3 (**b**), OsEULD1A_1 (**c**), OsEULD1A_2 (**d**), OsEULD1B_1 (**e**), OsEULD1B_2 (**f**), OsEULD2_1 (**g**), and OsEULD2_2 (**h**). Beta-sheets, stretches of α-helices and loops are colored magenta, orange and green, respectively. The bundles of β-sheet are numbered I, II and III, respectively. The carbohydrate-binding site of the EUL domain is located at the bundle III. **i**-**j**. Ribbon structure of the two-β-trefoil domain OsEULD1A (**i**) and OsEULD1B (**j**), showing the respective arrangement of the two β-trefoil domains on both sides of the central linker region (L). The arrow indicates the carbohydrate-binding site in subdomain III. N and C correspond to the N- and C-terminal ends of the polypeptide chain of OsEULD1A and OsEULD1B, respectively

Molecular modeling of the EUL domains revealed that they all consist of the canonical β-trefoil conformation found in bacterial lectins, made of three bundles of β-strands, labelled as subdomains I, II and III, linked by more or less extended loops which protrude out of the protein core (Fig. 3a-h). A single functional carbohydrate-binding site was identified in subdomain III, whereas three functional carbohydrate-binding sites usually occur in the bacterial β-trefoil structures. Modeling of the D-type lectins, OsEULD1A, OsEULD1B and OsEULD2, revealed that they consist of two tandemly arrayed β-trefoil domains, linked by a more or less extended proline (P)-rich linker region (Fig. 3i-j). Each β-trefoil domain in the lectin contains one carbohydrate-binding site which upon folding is located at opposite ends of the polypeptide chain.

**Fig. 4:**
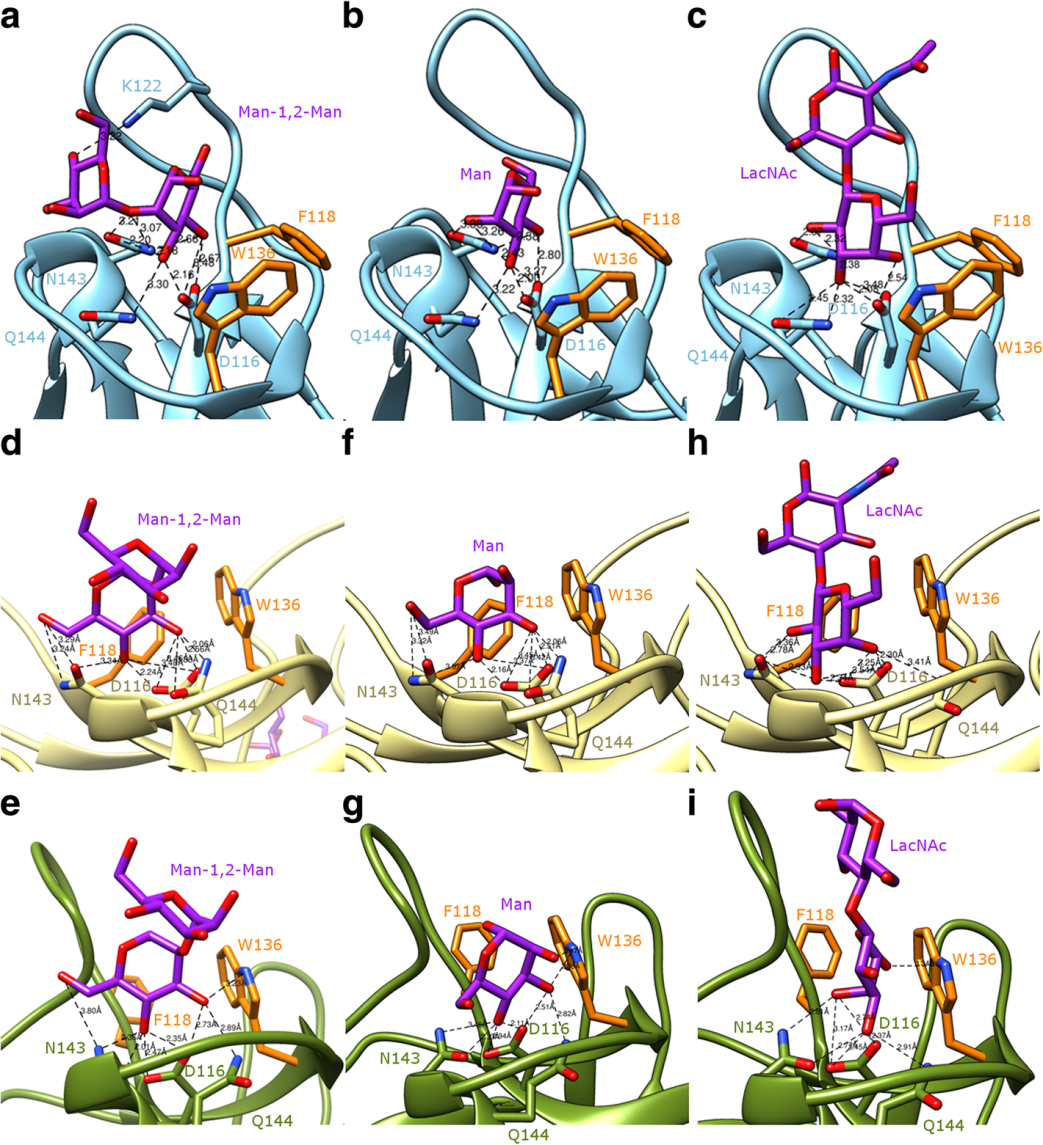
Binding models of selected ligands in the carbohydrate-binding site of OsEULS2 and OsEULD1A. A-C. Side-view of the carbohydrate-binding site of OsEULS2 showing the docking of Man1,2Man (**a**), Man (**b**) or LacNAc (**c**) to the carbohydrate-binding site. **d**-**i**. Side-view of the carbohydrate-binding site of OsEULD1A showing the docking of Man1,2Man (**d**-**e**), Man (**f**-**g**) or LacNAc (**h**-**i**) to the carbohydrate-binding site of the first (**d**, **f** and **h**) or second (**e**, **g** and **i**) EUL domain. The H-bond distances are indicated (Å). A stacking interaction occurs between the first Man ring (**a**, **d** and **e**), Man (**b**, **f** and **g**) or Gal (**c**, **h** and **i**) and the aromatic residues F118 and W136 (colored orange) located in the vicinity of the active site

As previously reported by Fouquaert and Van Damme (2012) both EEA and OsEULS2 exhibit a high specificity towards mannose and galactose containing saccharides. Docking experiments performed with α-D-mannose (Man), α-1,2-dimannoside (Man1,2Man), and N-acetyl-D-lactosamine (LacNAc), confirmed the promiscuity of the carbohydrate-binding site of the single-domain lectins OsEULS2 and OsEULS3 (Fig. 4, Additional file 5: Figure S2). A network of 8–9 hydrogen bonds participate in the anchorage of Man or Gal to the D116, N143 and Q144 residues forming the functional triad of the carbohydrate-binding site. An additional hydrogen bond occurs between K122 of OsEULS2 and the second Man ring of Man1,2Man. Additional stacking interactions between the sugar rings and the aromatic residues F118 and W136, complete the binding of simple sugars and disaccharides to the carbohydrate-binding site. Because of the high sequence conservation for the residues forming the active triad D-N-Q of EULs (Fig. 2b) a very similar binding scheme was observed in docking experiments performed with the two-domain lectin OsEULD1A (Fig. 4) and OsEULD2 (Additional file 6: Figure S3). Depending on the lectins, some discrepancies were noticed in the binding of simple sugars to the carbohydrate-binding site. These discrepancies mainly concern 1) the number and the length of hydrogen bonds connecting the sugar rings to the D116-N143-Q144 triad and, 2) the stacking interactions between the sugar rings and the aromatic residues F118 and W136. In this respect, the aromatic residue F118 is replaced by the hydrophobic residue L118 in both OsEULS3 (Additional file 6: Figure S2) and the second EUL domain of OsEULD1B, preventing a stacking interaction with the Man or Gal ring. In spite of these few discrepancies, the binding scheme of Man and Gal remains very similar from one to another EUL domain.

### Expression profiles of EUL genes upon plant stress

**Fig. 5:**
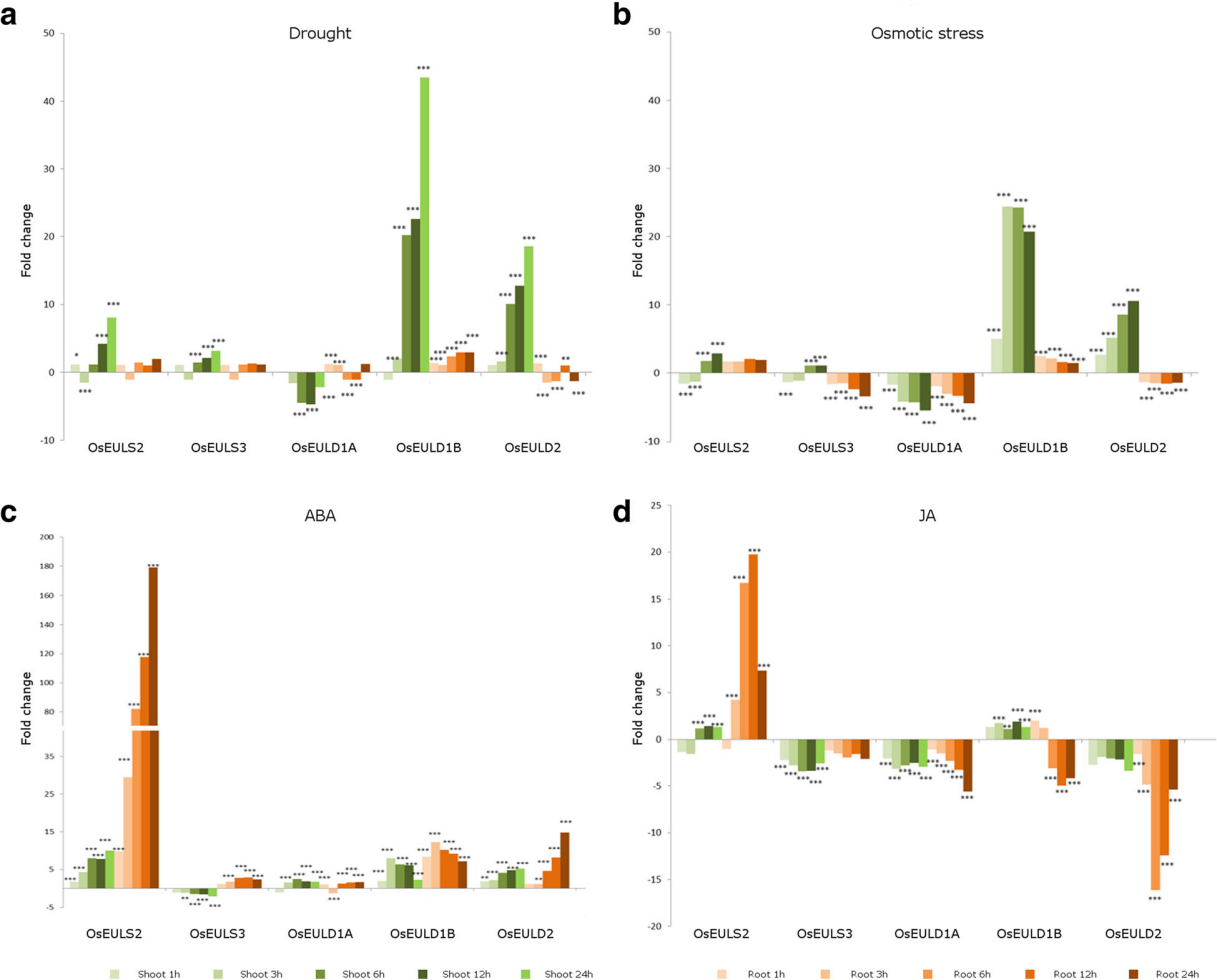
Expression profiles of the rice EUL genes upon plant stress represented as fold change. The false discovery rate is represented with asterisks to denote statistical significance. One star (*) is used if the value is less than 0.05, two stars (**) represent values less than 0.05 but higher or equal to 0.001, and three stars (***) show a value below 0.001. **a**. Drought stress. **b**. Osmotic stress. **c**. ABA treatment. **d**. JA treatment

A search in the Transcriptome Encyclopedia of Rice (TENOR) database (Kawahara et al. 2016) was performed and changes in the expression levels were observed for the EUL genes after different abiotic stresses and plant hormone treatments. Figure 5 shows the expression profiles for the different OsEUL genes based on the expression data after application of drought or osmotic stress as well as after exogenous application of absiscic acid (ABA, 100 μM) or jasmonic acid (JA, 100 μM).

Transcripts for OsEULD1B and OsEULD2 are highly and significantly upregulated in the shoots in response to drought and osmotic stress (43 and 24 times for OsEULD1B, respectively, and 19 and 11 times for OsEULD2, respectively), especially after 3–12 h of treatment. Though the expression of OsEULS2 and OsEULS3 was also increased after drought stress the fold change is only eight times for OsEULS2 and three times for OsEULS3 after one day of treatment which is significantly lower in comparison with OsEULD1B and OsEULD2. The effect from the osmotic stress on the expression of the S-type EULs in the rice shoots is not so prominent, being only three and one times for OsEULS2 and OsEULS3, respectively. Upregulation of OsEULD1B and OsEULS2 was also observed in the roots whereas transcript levels for OsEULD2 were downregulated. Similarly transcript levels for OsEULS3 were downregulated in roots subjected to osmotic stress. Expression of OsEULD1A was significantly downregulated in shoots and roots after drought and osmotic stress.

Upon hormone treatment with ABA and JA, OsEULS2 is highly upregulated, especially in roots. Transcript levels for all other OsEULs were also significantly changed in shoots and roots, except for a slight downregulation in shoots for OsEULS3. In contrast to ABA, JA mainly causes a downregulation for all EULs with exception of OsEULS2 (up to 20 fold upregulation in roots) and OsEULD1B (5 fold upregulation in shoots).

### Cis-regulatory elements in EUL promoter sequences

**Fig. 6:**
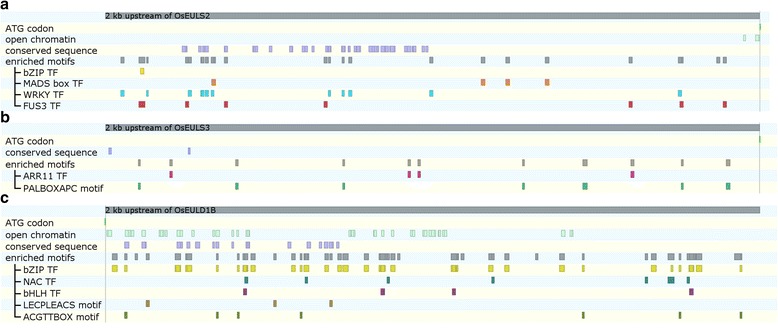
Cis-regulatory elements in the EUL promoter sequences. Elements identified in the OsEULS2 (**a**), OsEULS3 (**b**) and OsEULD1B (**c**) promoter by the different analyses performed in the integrated approach: motifs in open chromatin, motifs in conserved sequences and motifs enriched in OsEUL regulons. The motifs enriched in the OsEUL regulons are annotated according to the associated transcription factor or motif name. Gray bars represent the 2.000 bp upstream from the ATG codon of the different OsEULs

The identification of cis-regulatory elements in the promotor sequences of the OsEUL genes can provide information about environmental and developmental stimuli that might influence OsEUL gene expression which could support the expression data. Therefore 2000 bp promoter fragments for each OsEUL gene were screened for cis-regulatory elements. To decrease the high number of false positives associated with simple mapping of cis-regulatory elements and strongly increase the likelihood of retaining biologically functional elements, an integrative bioinformatics approach was followed which takes into account co-expressed genes, evolutionary sequence conservation and information on open chromatin regions (DNaseI-hypersensitive sites) (Vandepoele et al. 2009; Van de Velde et al. 2014). Cis-regulatory elements were identified in a set of approximately 150 co-expressed genes identified for each OsEUL gene. Furthermore an enrichment analysis was performed on this set of genes to identify the cis-regulatory elements significantly shared between each OsEUL and its co-expressed genes. In total 41 cis-regulatory elements were identified in the OsEUL promoters and their respective co-expressed genes, yielding regulatory information about OsEULS2, OsEULS3 and OsEULD1B (Fig. 6; Additional file 7: Table S4; Additional file 8: Figure S4). In addition, 111 cis-regulatory elements were identified in open chromatin regions of OsEULS2, OsEULD1A and OsEULD1B (Additional file 7: Table S4; Additional file 8: Figure S4) and 410 sites were identified within the evolutionary conserved regions of all OsEUL promoters (Additional file 7: Table S4; Additional file 8: Figure S4).

Analysis of the transcription factors binding to the identified cis-regulatory elements shared between the OsEULs and their co-expressed genes (OsEUL regulons) retrieved two major transcription factor families involved in stress response and development, in particular the WRKY family and the basic leucine-zipper (bZIP) family. While the WRKY transcription factors are mainly found in association with OsEULS2, the bZIP family as well as some NAC transcription factors are associated with OsEULD1B. Within the promoter sequence of OsEULS2, 78% of the significantly overrepresented motifs were identified as elements associated to the WRKY family of transcription factors (Fig. 6; Additional file 7: Table S4; Additional file 8: Figure S4). Our analysis retrieved binding sites for 5 rice WRKY transcription factors : WRKY11 (LOC_Os01g43650), WRKY28 (LOC_Os06g44010), WRKY62 (LOC_Os09g25070), WRKY71 (LOC_Os02g08440) and WRKY76 (LOC_Os09g25060). In addition, binding sites of five maize and one *Arabidopsis* WRKY transcription factor and one additional W-box motif were retrieved (Additional file 7: Table S4). Furthermore, binding sites for two bZIP transcription factors from *Arabidopsis* (bZIP19 and bZIP23) were identified. The other identified motifs in the OsEULS2 regulon are associated with FUS3 and MADS box transcription factors. The presence of a TATABOX1 motif was also confirmed in the OsEULS2 promoter.

Most of the significantly overrepresented motifs in the promoter sequence of the OsEULD1B gene belong to the so-called ABRE elements (e.g. ABRELATERD1, ABRETAEM, ABREBNNAPA, ABADESI2) or are associated with the bZIP family of transcription factors according to their annotation in the PLACE database (HY5AT, ACGTTBOX). In total, 59.4% of the significantly overrepresented motifs/transcription factors in the promoter sequence of the OsEULD1B gene are associated to bZIP transcription factors. Among these elements TRAB1, OSBZ8 and LOC_Os07g37920 represent bZIP transcription factors from rice. The second largest group are the NAC transcription factors which comprise 20.8% of the enriched promoter elements for OsEULD1B. Furthermore two binding sites for bHLH factors were retrieved. The motif enrichment analysis in the OsEULS3 regulon identified only two significantly enriched elements in the OsEULS3 promoter: a PALBOXAPC motif and a binding site for the *Arabidopsis* response regulator ARR11.

A GO term enrichment analysis was performed for the different sets of OsEUL regulons (Additional file 9: Table S5) and revealed several GO terms related to stress perception and signaling (Additional file 10: Table S6). For example, in the OsEULD1B regulon GO terms such as ‘ABA metabolism’ (GO:0009687), ‘response to abiotic stimuli’ (GO:0009628) and ‘abiotic stress’, ‘response to osmotic stress((GO:0006970), ‘response to salt stress’ (GO:0009651) and more general ‘response to stress’ (GO:0006950) and ‘response to stimulus’ (GO:0050896) were found to be enriched.

### Phylogenic relationships between the EUL domains from rice and other monocot and dicot species

While the occurrence of many lectin families is restricted to a few plant families, the EUL family is present in Embryophyta. However, differences in the number of EUL genes are observed between different plant species. Analysis of 8 monocot genomes retrieved 5 to 8 putative EUL genes (Additional file 11: Table S7). In dicot genomes, the number of putative EUL genes is remarkably smaller. Three EUL genes were identified in the genome of *Glycine max* (Van Holle and Van Damme 2015) while *Arabidopsis thaliana* (Van Hove et al. 2011) and *Cucumis sativus* (Dang and Van Damme 2016) only contain a single EUL gene. Further analysis of 5 other dicot genomes yielded 1 to 3 putative EUL genes (Additional file 11: Table S7). In addition, analysis of the domain structure of the EULs in dicots and monocots revealed that the D-type EULs are only present in monocots while the S-type EULs are found in all species (Additional file 11: Table S7).

**Fig. 7:**
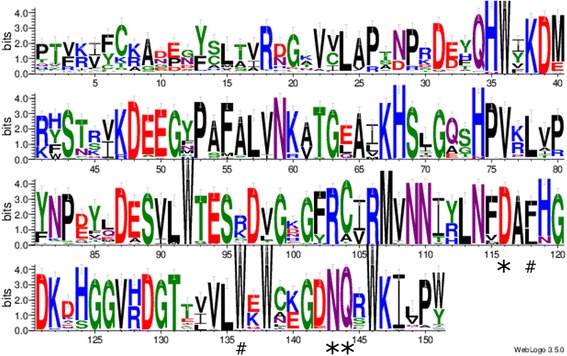
Sequence logo for the EUL domains of all species under study. Amino acid residues forming the active triad of the carbohydrate-binding site are indicated with an asterisk (*). Aromatic residues involved in stacking interactions with the sugars are indicated by a hash (#)

Since a high level of sequence conservation was observed between the different EUL domains in rice (Additional file 4: Table S3), this analysis was extended with the EUL domains obtained from the different monocot and dicot species. In total 70 EUL domains belonging to 33 S-type and 19 D-type EULs (Additional file 11: Table S7) were aligned and the variability in the amino acids at each position of the sequence was analyzed and quantified using WebLogo (Fig. 7). Despite variation in the first 33 amino acids of the EUL domain, a high level of conservation is observed starting from the first QxW motif (at position 34–36) in the EUL domain. The D116-N143-Q144 consensus sequence representing the functional triad of the carbohydrate-binding site was conserved in all analyzed sequences. Next to this triad, the aromatic residues involved in stacking interactions with the sugars are conserved. As expected from the modeling studies, the W136 residue was conserved in all species except for *Vitis vinifera* where an asparagine (N) is present, while for the residue F118 a substitution with the hydrophobic residue L118 was observed in 37% of the sequences.

**Fig. 8:**
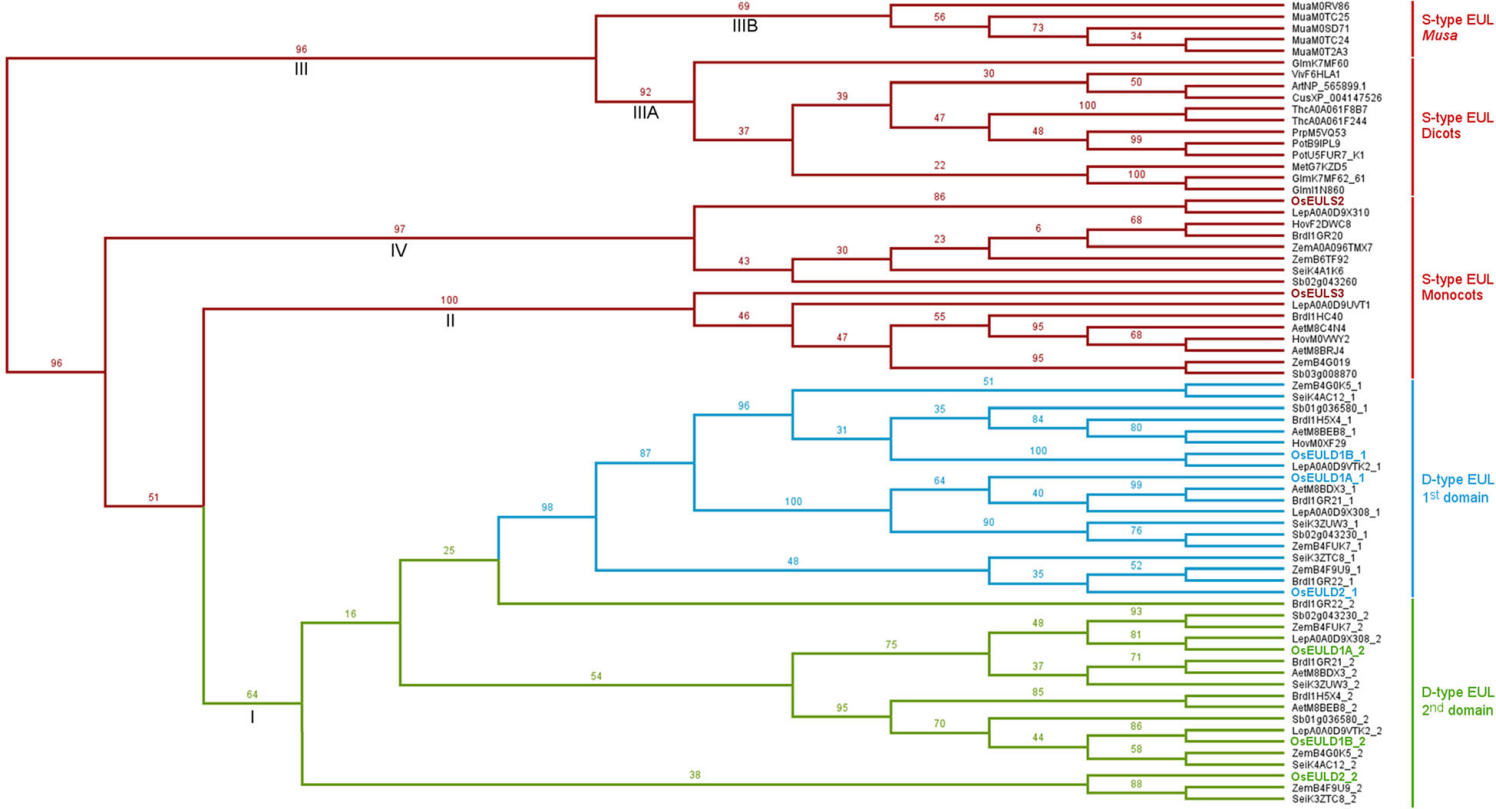
Phylogenetic tree for all EUL domains retrieved from 9 monocot and 8 dicot species. EUL domains are annotated as S-type (*red*) or D-type EUL domains. The D-type domains are subdivided in domain 1 (N-terminal EUL domain) (*blue*) and domain 2 (C-terminal EUL domain) (*green*). Branch labels show bootstrapping values

To study the evolutionary relationships between these EUL domains, a phylogenetic analysis was performed. All EUL domain sequences were aligned, after which a phylogenetic tree was generated (Fig. 8). The dendrogram shows 4 main branches (indicated as I to IV) and reveals a rather strict separation between the EUL domains originating from S-type and D-type EULs (Fig. 8). Within the cluster of D-type EUL domains (branch I), a separation can be observed between the N-terminal (domain 1) and C-terminal (domain 2) EUL domains. The branches show multiple clusters defined by the rice D-type EULs: OsEULD1A, OsEULD1B and OsEULD2 (Fig. 8). While the D-type EUL domains group closely together, the clustering of the S-type EUL domains shows more divergence.

The EUL domains from dicot species cluster together in one branch (branch IIIA) separately from the monocot species. In addition, the sequences from *Musa acuminata*, the only monocot with only S-type EULs, cluster together in one branch (branch IIIB) separated from the other monocots but close to the branch of the dicot EUL domains. For the other monocot S-type EUL domains two clusters are observed, these two clusters can be defined by OsEULS2 (branch IV) and OsEULS3 (branch II).

Reconciliating the domain tree with the species tree gave insights into the duplication events that gave rise to the S- and D-type EULs (Additional file 12: Figure S5). After the divergence between *Musa acuminata* and the *Poaceae*, the ancestral S-type was submitted to three consecutive duplication events. The first two duplication events gave rise to the two S-type EULs (represented by OsEULS2 and OsEULS3), the third duplication event gave rise to the 2 domains of the D-type EULs. Further duplication events yielded the different D-type EULs and increased the copy number of EULs in the different species. Increasing the domain tree with sequences from monocots outside the *Poaceae* family might increase the resolution of the timeframe when the different duplication events occurred.

## Discussion

With changing environmental conditions, the ability of plants to react to different forms of stress (e.g. cold, drought and flooding) becomes of key importance for a sustainable future. Understanding the mechanisms governing plant stress responses and identification of the individual components involved, will shed light on the molecular mechanisms behind stress signaling which in turn can be applied to improve stress resistance (Jung et al. 2013). Accumulating evidence shows that protein-carbohydrate interactions are of vital importance for plant immunity, involving lectin-carbohydrate interactions at the cell surface as well as in the cytoplasmic compartment (De Schutter and Van Damme 2015; Lannoo and Van Damme 2014).

Based on their sequence similarity to reference members for the different plant lectin families, we retrieved 325 putative lectin genes belonging to nine different lectin families from the genome of *Oryza sativa* subsp. japonica, indicating that the rice genome harbours a large set of putative lectin domains each characterized by a specific carbohydrate-recognition domain. Since experimental data points to the involvement of EULs in the plant stress response (Van Hove et al. 2015; Al Atalah et al. 2014a; Fouquaert et al. 2009), the focus of this paper is on an in-depth molecular characterization of this lectin family in rice.

Five EUL genes were identified in the genome of japonica rice. Orthologs of these 5 genes were also found in the indica genome (Additional file 11: Table S7). Since several databases provide evidence that these EUL genes are expressed, we assume that these five genes are functional. In addition, four pseudogenes have been reported (Fouquaert et al. 2009) for which no evidence of transcription was found. Although all four pseudogenes were retrieved from the japonica genome based on homology with the *E. europeaus* lectin (Additional file 11: Table S7), only two of them were found to contain an EUL domain using interproscan. Similarly, 2 pseudogenes containing EUL domains were identified in the subspecies indica. Since there is no evidence for transcription of these pseudogenes, the sequences were not taken into account for further analysis.

Analysis of the genomic structure of the rice EUL genes revealed a high degree of conservation. When plotting the intron-exon structure of the OsEUL genes, to the protein sequence, it is noted that exon boundaries are present at the same positions in the protein sequences. Furthermore, when analyzing the position of the different rice EULs in the genome, it is observed that OsEULD1A and OsEULD2 are linked by a tandem duplication, which can account for the high similarity in genomic structure. Interestingly, a high degree of similarity is also observed between the intron-exon structure of OsEULS3 and ArathEULS3, the only EUL gene in the *Arabidopsis thaliana* genome. Due to the similarity between the structure of ArathEULS3 and OsEULS3/D1A/D2, it is suggested that the S3 type represents the more ancestral structure.

A search in the SNPSeek database comprising the data of the 3000 rice genome project, identified 46 SNPs in the rice EUL genes. Analysis of the SNPs that give rise to an amino acid change revealed clear preferences between indica and japonica subspecies for the SNPs in the D-type EUL genes. Further research is needed to elucidate whether the SNPs contribute to differences in stress tolerance of rice.

In PFam (PF14200, Ricin-B lectin 2, 103 amino acids) and SSF (SSF30570, Ricin-B like lectins, 151 amino acids) the EUL domains are annotated as ricin-B like lectins based on the presence of the QxW motif typical for ricin-B domains. Compared to the shorter pFam domain, the annotation of the EUL domain by SSF (SSF50370) shows an extended domain of 151 amino acids including a second QxW motif. Since the EUL genes share no significant overall sequence similarity with any protein comprising a ricin-B domain (Fouquaert et al. 2008), lectins containing the EUL domain were grouped as a separate lectin family.

Phylogenetic analysis of the EUL domains showed a close relationship between the EUL domains of the S-type EULs and the second domain of the D-type EULs. The percentage sequence identity between the S-type EUL domain and domain 1 of D-type EULs ranges between 68 and 76% (similarity between 80 and 86%) while the sequence identity between S-type EUL domains and domain 2 of D-type EULs ranges between 71 and 80% (similarity between 82 and 92%) (Additional file 4: Table S3). This higher sequence similarity between the S-type EULs and the second domain in D-type EULs is in agreement with the higher degree of conservation in the genomic structure of these domains.

As pointed out by Fouquaert and Van Damme (2012), and documented by Agostino et al. (2015), the carbohydrate-binding domain of the *Euonymus* lectin exhibits a notable promiscuity in the accommodation of diverse carbohydrate structures. The spatial organization of the amino acid triad D-N-Q forming the carbohydrate-binding site of EULs, accounts for the promiscuity in the binding of simple sugars and oligosaccharides to the β-trefoil domains of *E. europaeus* lectins. In this respect, docking experiments performed with mannose, α-1,2-dimannoside and LacNAc, resulted in the anchorage of the sugars to the carbohydrate-binding site of the β-trefoil domains through a very similar network of hydrogen bonds and stacking interactions. However, some discrepancies occurred according to the number and length/strength of hydrogen bonds anchoring the sugars to the carbohydrate-binding site. The docking experiments with OsEULS2 confirmed the previously reported substrate specificity for high-mannose N-glycans and lactosamine related structures (Al Atalah et al. 2012). However, for OsEULD1A a substrate specificity for galactose related sugars or galactose containing glycoproteins was reported whereas mannose did not inhibit the agglutination of rabbit erythrocytes caused by OsEULD1A.

Previous promoter analysis performed using the PLACE database (Al Atalah et al. 2013) identified the presence of several TATA and CAAT boxes and identified three major classes of promoter elements, among which the light responsive elements, the ABA and GA responsive elements and the elements related to other (a)biotic stresses. There is some controversy about the significance of the TATA box for transcription initiation since analyses in *A. thaliana* (Molina and Grotewold 2005) and rice (Civan and Švec 2009) genomes revealed that only 29% of the Arabidopsis genes and 19% of the genes in *O. sativa* comprise a TATA box in their promoter region. The enrichment analysis identified one TATABOX1 motif in OsEULS2 promoter, but similar motifs were absent in other OsEUL promoter sequences. Identification of cis-regulatory elements revealed a high number of promoter elements putatively related with the abiotic stress responsiveness of the OsEUL genes. To discriminate between false positives and true biologically significant elements, coregulatory genes, evolutionary sequence conservation and information about open chromatin regions were integrated into the analysis. A large set of cis-regulatory elements retrieved from the OsEUL promoter sequences can be classified in the WRKY, basic leucine-zipper (bZIP) and NAC transcription factor families, known to be associated with abiotic stress responses (Banerjee and Roychoudhury 2017; Fang et al. 2008; Nijhawan et al. 2008). These data are in agreement with the GO enrichment analysis that yielded GO terms related to stress signaling and metabolism, response to abiotic stimuli and abiotic stress, cell signaling etc.

Motifs associated with WRKY transcription factors are abundantly present within the promoter sequences of the OsEULS2 regulon. WRKY transcription factors have been implicated in different biological processes such as response to wounding, senescence, development, dormancy, cold and drought tolerance, metabolism and hormone signaling pathways. In addition, numerous WRKY genes are involved in response to biotic and abiotic stress (Berri et al. 2009). The five rice WRKY transcription factors with binding sites identified in the OsEULS2 promoter all show stress regulated expression profiles. The responsiveness of WRKY11, WRKY28 and WRKY62 to abiotic stress was shown in a microarray analysis (Jain et al. 2007), where WRKY11 was upregulated in drought stress, whereas WRKY28 and WRKY62 were responsive to salt stress. With the exception of WRKY11, these transcription factors were also upregulated under several biotic stresses, such as bacterial, viral and fungal infection (Marcel et al. 2010; Zhou et al. 2010; Berri et al. 2009).

Within the promoter of OsEULS3, and its coregulated genes, a PALBOXAPC element was identified (Additional file 7: Table S4). This element was found to be significantly enriched in promoters of stress related genes that are downregulated by ABA (Yazaki and Kikuchi, 2005) and agrees with the downregulation of OsEULS3 in rice shoots treated with ABA (Fig. 5).

A predominant group of motifs in the OsEULD1B promoter are associated with transcription factors belonging to the bZIP family, these are known as stress inducible transcription factors (Banerjee and Roychoudhury 2017) involved in drought, osmotic, salt stress and some of them also in cold stress (Liu et al. 2014; Lu et al. 2009). Among them TRAB1 and OSBZ8 are reported as ABA related elements. While OSBZ8 expression is reported in the roots of ABA treated seedlings and in developing embryos, TRAB1 shows more general expression in different plant organs as well as in different stages of embryo development and in seeds (Banerjee and Roychoudhury 2017). Their responsiveness to dehydration and salt is confirmed in microarray data (Nijhawan et al. 2008) and in the case of OSBZ8 the upregulation is also associated with salt tolerance in indica rice cultivars (RoyChoudhury et al. 2008). Another group of transcription factors retrieved in the EULD1B promoter analysis belongs to the NAC transcription factor family, involved in different stress and developmental processes (Fang et al. 2008). Previously the NAC/NAM transcription factor LOC_Os07g37920 was identified as a stress inducible gene under water deficit conditions (Ray et al. 2011). Similarly Fang et al. (2008) reported upregulation of this transcription factor in response to salt and drought stress. RNA sequencing data showed that the same transcription factor was involved in root development and auxin signaling (Hiltenbrand et al. 2016).

Enhanced expression for OsEUL genes in plants subjected to abiotic stress was confirmed by proteomics data. Already in 1997 Moons et al. reported on the stress induced expression of one of the OsEULs after treatment of rice seedlings with ABA (Moons et al. 1997). More recent proteomic analyses (Cheng et al. 2009), qRT-PCR investigations (Al Atalah et al. 2014a) have confirmed the differential transcription of several OsEUL genes upon ABA and salt stress. Expression of OsEULD1B was found to be upregulated upon drought stress (Rabello et al. 2008). EUL proteins in barley and wheat were also reported as drought related proteins. EULS3 and EULD1B genes are induced and upregulated at protein level in barley crown and roots from drought sensitive cultivars while the drought tolerant cultivar shows a constant protein level for EULD1B (Vítámvás et al. 2015). Similarly, two proteins belonging to the EUL family, one of them classified as the EULD2 type are detected at higher protein levels in a drought tolerant wheat cultivar especially in drought treated plants compared to the control plants at 10 days after opening of the flowers (Jiang et al. 2012b).

Transcript levels for ArathEULS3, the EUL homolog from Arabidopsis were also significantly upregulated after ABA treatment (Li et al. 2014; Van Hove et al. 2014), MeJA treatment (Van Hove et al. 2014) and drought (Li et al. 2014). In addition, Arabidopsis plants overexpressing ArathEULS3 revealed a better tolerance to drought stress (Li et al. 2014) and *Pseudomonas syringae* infection (Van Hove et al. 2014). All these findings support the statement that EUL genes are actively involved in the signal cascades triggered by several abiotic stresses.

In contrast to ABA, JA causes a significant downregulation for most EULs in rice with the exception of OsEULS2 (shoots and roots) and OsEULD1B (mainly shoots). The information retrieved from TENOR is confirmed by proteomics studies involving some EULs (Cho et al. 2007). Moons et al. (1997) also report the downregulation of OsEULD1B in roots after 24 h after JA treatment which is in agreement with the RNA sequencing data and in the same paper EULD1B is defined as not responsive to salicylic acid (SA) or ethylene.

At present, there are only a few reports that show the involvement of OsEUL genes in response to pathogen attack. qRT-PCR experiments and microarray data revealed that transcript levels for some OsEULs are upregulated after infection of rice with *Xanthomonas oryzae* pv. oryzae (OsEULD1B, OsEULD2), *Magnaporthe oryzae* (OsEULD2) or root knot nematodes (*Meloidogyne graminicola*) (OsEULD2) (Al Atalah et al. 2013; Kyndt et al. 2012). In line with this, several WRKY transcription factors with binding sites identified in the OsEUL promoters are regulated by diverse biotic stresses. For example, WRKY71 for whom a binding site was identified in the OsEULS2 promoter was upregulated upon infection with *Magnaporthe grisea* (Berri et al. 2009). Other WRKY transcription factors with binding sites in the OsEULS2 promoter were upregulated upon infection with *Xanthomonas oryzae* (Zhou et al. 2010) and *Magnaporthe oryzae* (Marcel et al. 2010).

The EUL lectin family is unique in that representatives of this family are present throughout the plant kingdom and have been identified in every fully sequenced genome of land plants. However, differences are observed in the number of EUL genes and the type of EULs in different plant species. The analysis of 9 monocot and 8 dicot genomes revealed a higher number of putative EUL genes in monocots (ranging 5 to 8) compared to dicots (ranging 1 to 3). In addition, it is observed that dicot species only possess S-type EULs while S-type as well as D-type lectins are identified in monocots (Additional file 7: Table S4). One exception is *Musa acuminata* where only S-type EUL sequences were identified. Judging from 70 EUL domain sequences, representing 33 S-type and 19 D-type EULs, identified in all genomes under study, the conservation of the EUL sequences is high. A high degree of sequence conservation was observed not only for the D-N-Q triad and the aromatic residues F/L118-W136 but also for the complete EUL domain.

In addition, the phylogenetic tree built from all EUL domains under study, revealed distinct clusters. Overall a good correlation was observed between the annotation of the EUL domains for the different species and the rice domain in this cluster. Reconciliation with the species tree provided insight in the three duplication events leading to the different S-type and D-type EULs. Interestingly the EUL domains from the dicot species are grouped in a separate branch and this branch clusters close to the cluster containing the EUL domains from *Musa acuminata* which, like the dicots, solely encodes the S-type lectins. Analysis of the reconciliated tree revealed that *Musa acuminata* diverged from the other monocots before the first duplication event that gave rise to the monocot S- and D-type EULs. This can explain why banana only contains S-type EUL lectins of the same (ancestral) type as the dicot species. In other monocot species the S-type EULs were subject to differentiation that gave rise to the S2-type and S3-type EULs.

## Conclusion

The strong conservation of the *Euonymus*-related lectins within the plant kingdom, not only at protein level but also at genomic level suggests an important role for these proteins in the plant cell. The identification of multiple stress responsive elements in the promoter sequences of these genes as well as the transcriptional regulation of these genes in response to stress or hormonal treatments are in agreement with a role in plant stress signaling. However, further research, such as the generation of rice plants overexpressing OsEULs, is needed to experimentally prove the involvement of EULs in stress signaling and plant defense in rice.

## Methods

### Identification and annotation of lectins in the *O. sativa* genome

Protein sequences encoding the reference members of the different lectin families [*Agaricus bisporus* agglutinin (ABA), Q00022.3; *Amaranthus caudatus* agglutinin (amaranthin), AAL05954.1; *Robinia pseudoacacia* chitinase-related agglutinin (CRA), ABL98074.1; *Nostoc ellipsosporum* agglutinin (cyanovirin), P81180.2; *Euonymus europaeus* agglutinin (EUL), ABW73993.1; *Galanthus nivalis* agglutinin (GNA), P30617.1; *Hevea brasiliensis* agglutinin (hevein), ABW34946.1; *Artocarpus integer* agglutinin (JRL), AAA32680.1; *Glycine max* agglutinin (legume lectin), P05046.1; *Brassica juncea* LysM domain (LysM), BAN83772.1; *Nicotiana tabacum* agglutinin (nictaba), AAK84134.1; *Ricinus communis* agglutinin lectin chain (ricin-B), 2AAI_B] were used to perform BLAST searches against the *Oryza sativa* subsp. japonica genome (RGAP release 7) available from NCBI (https://blast.ncbi.nlm.nih.gov), MSU (Kawahara et al. 2013) and phytozome (https://phytozome.jgi.doe.gov), as described previously by Van Holle and Van Damme (2015). Top hits were used for a consecutive BLAST search. In addition the MSU database (Kawahara et al. 2013) was searched using the Pfam domain identifier [ABA: PF07367 (fungal fruit body lectin); amaranthin: PF07468 (agglutinin domain); CRA: PF00704 (glycol-hydro 18); cyanovirin: PF08881 (CVNH); EUL: PF14200 (ricin-lectin 2); GNA: PF01453 (B-lectin); hevein: PF00187 (chitin bind 1); JRL: PF01419 (jacalin); legume lectin: PF00139 (lectin legB); LysM: PF01476 (LysM domain); nictaba: PF14299 (PP2); ricin-B: PF00652 (ricin-B lectin)] of the different lectin domains. Protein sequences were downloaded from MSU (Kawahara et al. 2013) and screened for the presence of conserved protein domains using interproscan 5 (Mitchell et al. 2015). The program was downloaded (https://www.ebi.ac.uk/interpro/download/) and locally installed. Indica lectins were identified by BLAST searches with the lectin domains of the japonica hits against the indica rice genome (ASM465v1) available from EnsemblPlants (http://plants.ensembl.org). As for the japonica sequences, these protein sequences were analyzed for the presence of conserved protein domains using Interproscan 5 (Mitchell et al. 2015). Only sequences with at least one lectin domain were retained. The protein sequences of the lectins were analyzed for the presence of signal peptides using SignalP 4.1 (Petersen et al. 2011) and the presence of transmembrane domains was analyzed using TMHMM 2.0 (Krogh et al. 2001).

### Mapping lectin sequences to chromosomal locations and analysis of duplication events

The putative lectin genes were mapped on the different chromosomes using the MapChart software (Voorrips 2002). The transcription start sites of the putative lectin genes were retrieved from the MSU database (Kawahara et al. 2013) and used for the construction of the map.

Gene expansion through segmental duplication or tandem duplication was analyzed for the japonica lectin sequences. Tandem duplications were assigned if: 1. Both genes belong to the same lectin family, 2. There are no more than ten intervening genes and 3. They reside on the same chromosome. Segmental duplications were identified using the Plant Genome Duplication Database (PGDD) (Lee et al. 2013). Collinear blocks within the *O. sativa* subsp. japonica genome were determined by McScan v8 (http://chibba.agtec.uga.edu/duplication/index/files), the output data was downloaded and searched for presence of lectin genes. Duplicated genes with a Ks (synonymous substitution) value higher than 1.0 were omitted.

### Genomic analysis of the *O. sativa* EUL genes

The genomic structure of the OsEUL genes was analyzed in GenomeView (Abeel et al. 2011) using the japonica RGAP release seven sequence. Visual representation of the genomic structure of the EUL genes was made in IBS 1.0.1 (http://ibs.biocuckoo.org/). Sequences of the intron boundaries were analyzed by Weblogo (http://weblogo.berkeley.edu/logo.cgi).

Single nucleotide polymorphisms (SNP) in the EUL genes between different rice subspecies were identified using the SNP-Seek database containing SNP genotyping data (called against Nipponbare reference Os-Nipponbare-Reference-IRGSP-1.0) from the 3000 Rice Genomes Project (Alexandrov et al. 2015). The use of the Nipponbare reference allele in the different rice groups (in %) is represented in a heat map using the BAR HeatMapper Plus Tool (http://bar.utoronto.ca/ntools/cgi-bin/ntools_heatmapper_plus.cgi). Based on this data a dendrogram is constructed using DendroUPGMA (http://genomes.urv.cat/UPGMA/). For the generation of the dendrogram, the RMSD (Root Mean Square Deviation) distance coefficient has been used to compare between sets of variables.

### Molecular modeling of OsEULs

Homology modeling of the β-trefoil domains of EULs was performed with the YASARA Structure program (Krieger et al. 2002), running on a 2.53 GHz Intel core duo Macintosh computer. Up to eighteen different models were built up for each of the EUL domains corresponding to LOC_Os07g48500.1 (OsEULS2), LOC_Os01g01450.1 (OsEULS3), LOC_Os07g48490.2 (OsEULD1A), LOC_Os03g21040.2 (OsEULD1B), and LOC_Os07g48460.1 (OsEULD2) sequences, using the X-ray coordinates of different bacterial hydrolases and lectins as templates: a β-l-arabinopyranosidase from *Streptomyces avermitilis* (PDB code 3A21) (Ichinose et al. 2009), the HA-33/HA-17 hemagglutinin complex of *Clostridium botulinum* (PDB code 5B2H) (Sagane et al. 2016), the mosquitocidal holotoxin from *Bacillus sphæricus* (PDB code 2VSE) (Treiber et al. 2008), a computationally reconstructed β-trefoil subdomain module (PDB code 3PG0) (Broom et al. 2012), the HA33 neutotoxin from *Clostridium botulinum* (PDB code 4OUJ) (Lee et al. 2014), the 1,3Gal43A exo-β-1,3-galactanase from *Clostridium thermocellum* (PDB code 3VSF) (Jiang et al. 2012a), the β-trefoil lectin HA33/C from *Clostridium botulinum* type C neurotoxin (PDB code 3AJ5, 3AJ6) (Nakamura et al. 2011), the retaining xylanase from *Streptomyces olivaceoviris* E-86 (PDB code 2D24) (Suzuki et al. 2009), a family ten xylanase from *Streptomyces olivaceoviridis* E-86 (PDB code 1V6W) (Fujimoto et al. 2004), and the hemagglutinin of *Clostridium botulinum* (PDB code 3WIN) (Amatsu et al. 2013). These templates were selected based on the best BLAST E-values found for the amino acid sequences matching with the query sequence, using a cutoff value of 0.5 for the alignment score. Finally, a single hybrid model was built up, from the different previous models combining the best refined regions, for each of the EUL domains. In addition, a tentative modeling of OsEULD1A and OsEULD1B proteins corresponding to LOC_Os07g48490.2 and LOC_Os03g21040.2 sequences, was performed with the same templates to get an insight into the molecular organization of these two-domain lectins. Similarly, a single hybrid model was built up from the different previous models for these two OsEULs. PROCHECK (Laskowski et al. 1993) and ANOLEA (Melo and Feytmans 1998) were used to assess the geometric and thermodynamic qualities of the three-dimensional models (Additional file 13: Table S8). Using ANOLEA to evaluate the models, some residues of the EUL models exhibited an energy over the threshold value. These residues are mainly located in the loop regions, especially those loops connecting the β-sheets in the models. However, the calculated QMEAN6 score (Benkert et al. 2011; Arnold et al. 2006) of all of the models gave values > 0.5 (results not shown).

Docking of α-D-mannose (Man), α-1,2-dimannoside (Man1,2Man), and N-acetyl-D-lactosamine (LacNAc), to the carbohydrate-binding site of the β-trefoil domain of EULs, was performed with the YASARA structure program. Some docking experiments were performed at the SwissDock web server (http://www.swissdock.ch) (Grosdidier et al. 2011) as a control for our docking experiments. Molecular cartoons were drawn with Chimera (Pettersen et al. 2004).

### Expression analysis of EUL genes based on publicly available resources

The accession numbers of the representative transcripts of OsEULs were downloaded from Rice Annotation Project Data Base (RAP DB) (http://rapdb.dna.affrc.go.jp/). A search for changes in the expression of all rice EULs transcripts was performed in TENOR database (http://tenor.dna.affrc.go.jp/). Data for the fold change after drought, osmotic stress, abscisic acid (ABA) and jasmonic acid (JA) were downloaded and represented graphically. During our search in TENOR we found three representative transcripts for OsEULS3 and two for OsEULD1A. Since all transcripts for each of these OsEULs showed very similar results, their fold change was represented as mean values.

### Promoter sequence analysis

Cis-regulatory elements were obtained from AGRIS (Palaniswamy et al. 2006), PLACE (Higo et al. 1999), Athamap (Steffens et al. 2004), CisBP (Weirauch et al. 2014), JASPAR (Mathelier et al. 2015) and TRANSFAC (Matys et al. 2003). These elements were mapped to 2 kb upstream promoter regions of all genes for *Oryza sativa* MSU RGAP 7 (Kawahara et al. 2013) using cluster-buster with –c option set to 0 (Frith et al. 2003).

In order to reduce the high false positive rates associated with inferring regulatory interactions based on simple motif mapping, two approaches were used. A first approach consisted of filtering motif matches using cross-species sequence conservation or open chromatin regions. Therefore, motif matches were filtered using all 4 sets of conserved non-coding sequences from De Witte et al. (http://bioinformatics.intec.ugent.be/blsspeller/) (De Witte et al. 2015) and DNaseI-hypersensitive sites downloaded from Zhang et al. (2012). Only the motif matches that overlap by 50% with the above functional regions (open chromatin or conserved non-coding sequences) were retained (using intersectBed –f 0.5 from BEDTools; Quinlan and Hall 2010).

For the second approach motif enrichment analysis was used. Motif mapping information was combined with a set of up to 200 co-expressed genes (according their Pearson correlation coefficient)from the TENOR database (Kawahara et al. 2016). With the help of the RAP-DB ID convertor tool (http://rapdb.dna.affrc.go.jp/tools/converter) the RAP DB identifiers were converted to MSU locus numbers and used for the subsequent motif enrichment analysis. During the process of conversion some of the annotated RAP DB genes were not identified in the MSU database which reduced the number of co-expressed genes to approximately 150 genes for each OsEUL gene. Motif enrichment on these co-expressed gene was determined using the hypergeometric test with false discovery rate correction. Only significantly (q-value < 0.05) enriched motifs also present in the OsEUL promoters were retained as enriched motifs. The GO enrichment analysis on the different OsEUL regulons was performed using the GO enrichment tool in the PLAZA workbench of PLAZA 3.0 Monocots (Proost et al. 2015).

### Phylogenetic analysis

Sequences corresponding to the different EUL domains of the OsEULs were extracted and aligned with MUSCLE using the default settings (Edgar 2004). Based on this alignment a maximum likelihood phylogenetic tree was build using RAxML v8.2.4. RAxML used the GTRGAMMA model with automated determination of the best amino acid substitution model (i.e. the model with the highest likelihood score on the starting tree), random number seed and distinct starting trees. Bootstrap iterations to assess the robustness of the generated trees were decided automatically by RAxML. The phylogenetic tree was displayed with FigTree v1.4.2 (http://tree.bio.ed.ac.uk/software/figtree).

To extend the phylogenetic analysis to other species, putative EUL sequences were identified by BLAST searches with the lectin domains of the japonica hits against the genomes of 8 monocot and 7 dicot species available from EnsemblPlants (http://plants.ensembl.org). The monocots included are *Aegilops tauchii* (ASM34733v1), *Brachypodium distachyon* (v1.0), *Hordeum vulgare* (barley) (ASM32608v1), *Leersia perrieri* (Lperr_V1.4), *Musa acuminata* (banana) (MA1), *Setaria italic* (JGIv2.0), *Sorghum bicolor* (Sorbi1) and *Zea mays* (corn) (AGPv4). Dicots include *Arabidopsis thaliana* (TAIR10), *Glycine max* (soybean) (V1.0), *Medicago truncatula* (MedtrA17_4.0), *Populus trichocarpa* (poplar) (JGI2.0), *Prunus persica* (peach) (Prupe1_0), *Theobroma cacao* (cacao) (Theobroma_cacao_20110822) and *Vitis vinifera* (grape) (IGGP_12x). The putative EUL lectin sequence from *Cucumis sativus* (cucumber) was provided by Dang and Van Damme (2016). These protein sequences were analyzed for the presence of conserved protein domains using Interproscan 5 (Mitchell et al. 2015). Proteins with an annotated EUL domain were retained for further analysis. Sequences with a truncated EUL domain were omitted from the analysis.

The EUL domain of all putative EUL sequences from the monocot and dicot species under study was extracted and aligned with MUSCLE using the default settings (Edgar 2004). A logo of the EUL domain was generated with WebLogo 3 (http://weblogo.threeplusone.com/create.cgi). From the aligned sequences a maximum likelihood phylogenetic tree was built with RAxML v8.2.4 as described before. The FigTree v1.4.2 software was used to visualize and edit the phylogenetic tree. Reconciliation of the phylogenetic tree with the species tree was performed in Notung 2.9 (Stolzer et al. 2012). The species tree, containing all species from which EUL domains were sampled, was constructed in NCBI taxonomy (https://www.ncbi.nlm.nih.gov/Taxonomy/CommonTree/wwwcmt.cgi).

## Additional files

Additional file 1: Table S1. Sizes of intron and exon sequences for the *O. sativa* EUL genes. (XLSX 9 kb)

Additional file 2: Figure S1. Exon structure plotted on the protein sequences of the rice EULs. Sequences encoded by different exons are shown in different colors, the sequence corresponding to the EUL domain (pFam) is underlined. (PNG 1719 kb)

Additional file 3: Table S2. SNPs in *O. sativa* EUL exons. (XLSX 10 kb)

Additional file 4: Table S3. Identity and similarity between the different *O. sativa* EUL domains (% identity/% similarity). (XLSX 10 kb)

Additional file 5: Figure S2. Binding models of selected ligands in the carbohydrate-binding site of OsEULS3. A-C. Side-view of the carbohydrate-binding site of OsEULS3 showing the docking of Man1,2Man (A), Man (B) or LacNAc (C) to the carbohydrate-binding site. The H-bond distances are indicated (Å). A stacking interaction occurs between the first Man ring (A), Man (B) or Gal (C) and the aromatic residues F118 and W136 (colored orange) located in the vicinity of the active site. (PNG 457 kb)

Additional file 6: Figure S3. Binding models of selected ligands in the carbohydrate-binding site of OsEULD2. A, C and E. Side-view of the carbohydrate-binding site of OsEULD2_1 showing the docking of Man1,2Man (A), Man (C) or LacNAc (E) to the carbohydrate-binding site. A stacking interaction occurs between the first Man ring (A), Man (C) or Gal (E) and the aromatic residues F118 and W136 (colored orange) located in the vicinity of the active site. B, D and F. Side-view of the carbohydrate-binding site of OsEULD2_2 showing the docking of Man1,2Man (B), Man (D) or LacNAc (F) to the carbohydrate-binding site. A stacking interaction occurs between the first Man ring (B), Man (D) or Gal (F) and W136 (colored orange) located in the vicinity of the active site. The H-bond distances are indicated (Å). (PNG 853 kb)

Additional file 7: Table S4. Cis-regulatory elements in OsEUL promoter sequences. (XLSX 50 kb)

Additional file 8: Figure S4. Overview of different cis-regulatory elements mapped on the rice lectin genes. Elements identified in the promoters by the different analyses performed in the integrated approach: motifs filtered with conserved non-coding sequences (track A), motifs filtered with open chromatin (track B) and motifs enriched in co-expressed genes of lectin genes (track C). (PNG 191 kb)

Additional file 9: Table S5. GO enrichment analysis on EUL regulons. (XLSX 163 kb)

Additional file 10: Table S6. Stress related GO terms in the GO enrichment analysis. (XLSX 11 kb)

Additional file 11: Table S7. Accession numbers of the different EUL sequences. (XLSX 11 kb)

Additional file 12: Figure S5. Reconciliated tree. Phylogenetic tree of the EUL domains reconciliated with the species tree using Notung 2.9 (Stolzer et al. 2012). Duplication events are indicated with “D”. (PNG 19 kb)

Additional file 13: Table S8. Geometric and thermodynamic qualities of the beta-trefoil domains of EUL models built by homology modeling. (XLSX 9 kb)

## Abbreviations

CRA: *Robinia pseudoacacia chitinase-related agglutinin*
EEA: *Euonymus europeaus agglutinin*
EUL: *Euonymus* Lectin
GNA: *Galanthus nivalis* agglutinin

## References

[CR1] Abeel T, Van Parys T, Saeys Y, Galagan J, Van de Peer Y (2011). GenomeView: a next-generation genome browser. Nucl Acids Res.

[CR2] Agostino M, Velkov T, Dingian T, Willians SJ, Yuriev E, Ramsland PA (2015). The carbohydrate-promiscuity of *Euonymus europaeus* lectin is predicted to involve a single binding site. Glycobiol.

[CR3] Al Atalah B, Rougé P, Smith DF, Proost P, Lasanajak Y, Van Damme EJM (2012). Expression analysis of a type S2 EUL-related lectin from rice in *Pichia pastoris*. Glycoconj J.

[CR4] Al Atalah B, Fouquaert E, Van Damme EJM (2013). Promoter analysis for three types of EUL-related rice lectins in transgenic Arabidopsis. Plant Mol Biol Rep.

[CR5] Al Atalah B, De Vleesschauwer D, Xu J, Fouquaert E, Höfte M, Van Damme EJM (2014a) Transcriptional behavior of EUL-related rice lectins toward important abiotic and biotic stresses. J Plant Physiol 171:986-992 10.1016/j.jplph.2014.04.004 24974324

[CR6] Alexandrov TS, Wang W, Mansueto L, Palis K, Fuentes RR, Ulat VJ, Chebotarov D, Zhang G, Li Z, Mauleon R, Hamilton RS, McNally KL (2015). SNP-Seek database of SNPs derived from 3000 rice genomes. Nucl Acids Res.

[CR7] Amatsu S, Sugawara Y, Matsumura T, Kitadokoro K, Fujinaga Y (2013). Crystal structure of Clostridium botulinum whole hemagglutinin reveals a huge triskelion-shaped molecular complex. J Biol Chem.

[CR8] Arnold K, Bordoli L, Kopp J, Schwede T (2006). The SWISS-MODEL workspace: a web-based environment for protein structure homology modelling. Bioinformatics.

[CR9] Banerjee A, Roychoudhury A (2017). Abscisic-acid-dependent basic leucine zipper (bZIP) transcription factors in plant abiotic stress. Protoplasma.

[CR10] Benkert P, Biasini M, Schwede T (2011). Toward the estimation of the absolute quality of individual protein structure models. Bioinformatics.

[CR11] Berri S, Abbruscato P, Faivre-Rampant O, Brasileiro ACM, Fumasoni I, Satoh K, Kikuchi S, Mizzi L, Morandini P, Enrico Pè M, Piffanelli P (2009) Characterization of WRKY co-regulatory networks in rice and Arabidopsis. BMC Plant Biol 9:1–22. doi:10.1186/1471-2229-9-120. 10.1186/1471-2229-9-120 PMC2761919 19772648

[CR12] Broom A, Doxey AC, Lobsanov YD, Berthin LG, Rose DR, Howell PL, McConkey BJ, Meiering EM (2012). Modular evolution and the origins of symmetry: reconstruction of a three-fold symmetric globular protein. Structure.

[CR13] Cheng Y, Qi Y, Zhu Q, Chen X, Wang N, Zhao X, Chen N, Cui X, Xu L, Zhang W (2009). New changes in the plasma-membrane-associated proteome of rice roots under salt stress. Proteomics.

[CR14] Cho K, Agrawal GK, Shibato J, Jung YH, Kim YK, Nahm BH, Jwa NS, Tamogami S, Han O, Kohda K, Iwahashi H, Rakwal R (2007) Survey of differentially expressed proteins and genes in jasmonic acid treated rice seedling shoot and root at the proteomics and transcriptomics levels. J Proteome Res. 6:3581–3603. 10.1021/pr070358v 17711327

[CR15] Civan P, Švec M (2009). Genome-wide analysis of rice (*Oryza sativa* L. subsp. japonica) TATA box and Y patch promoter elements. Genome.

[CR16] Dang L, Van Damme EJM (2016). Genome-wide identification and domain organization of lectin domains in cucumber. Plant Physiol Biochem.

[CR17] De Schutter K, Van Damme EJM (2015). Protein-carbohydrate interactions as part of plant defense and animal immunity. Molecules.

[CR18] De Witte D, Van de Velde J, Decap D, Van Bel M, Audenaert P, Demeester P, Dhoedt B, Vandepoele K, Fostier J (2015). BLSSpeller: exhaustive comparative discovery of conserved cis-regulatory elements. Bioinformatics.

[CR19] Edgar RC (2004). MUSCLE: multiple sequence alignment with high accuracy and high throughput. Nucl Acids Res.

[CR20] Fang Y, You J, Xie K, Xie W, Xiong L (2008). Systematic sequence analysis and identification of tissue-specific or stress-responsive genes of NAC transcription factor family in rice. Mol Genet and Genomics.

[CR21] Fouquaert E, Van Damme EJM (2012). Promiscuity of the Euonymus carbohydrate-binding domain. Biomolecules.

[CR22] Fouquaert E, Peumans WJ, Smith DF, Proost P, Savvides SN, Van Damme EJM (2008). The “old” *Euonymus europaeus* agglutinin represents a novel family of ubiquitous plant proteins. Plant Physiol.

[CR23] Fouquaert E, Peumans WJ, Vandekerckhove T, Ongenaert M, Van Damme EJM (2009). Proteins with an Euonymus lectin-like domain are ubiquitous in Embryophyta. BMC Plant Biol.

[CR24] Frith MC, Li MC, Weng Z (2003). Cluster-Buster: Finding dense clusters of motifs in DNA sequences. Nucl Acid Res.

[CR25] Fujimoto Z, Kaneko S, Kuno A, Kobayashi H, Kusakabe I, Mizuno H (2004). Crystal structures of decorated xylooligosaccharides bound to a family 10 xylanase from *Streptomyces olivaceoviridis* E-86. J Biol Chem.

[CR26] Garcia-Vallve S, Palau J, Romeu A (1999). Horizontal gene transfer in glycosyl hydrolases inferred from codon usage in *Escherichia coli* and *Bacillus subtilis*. Mol Biol Evol.

[CR27] Grosdidier A, Zoete V, Michielin O (2011). SwissDock, a protein-small molecule docking web service based on EADock DSS. Nucl Acids Res.

[CR28] Higo K, Ugawa Y, Iwamoto M, Korenaga T (1999). Plant cis-acting regulatory DNA elements (PLACE) database: 1999. Nucl Acid Res.

[CR29] Hiltenbrand R, Thomas J, McCarthy H, Dykema KJ, Spurr A, Newhart H, Winn ME, Mukherjee A (2016). A developmental and molecular view of formation of auxin-induced nodule-like structures in land plants. Front Plant Sci.

[CR30] Hu C, Shi J, Quan S, Cui B, Kleessen S, Nikoloski Z, Tohge T, Alexander D, Guo L, Lin H, Wang J, Cui X, Rao J, Luo Q, Zhao X, Fernie AR, Zhang D (2014). Metabolic variation between japonica and indica rice cultivars as revealed by non-targeted metabolomics. Sci Rep.

[CR31] Ichinose H, Fujimoto Z, Honda M, Harazono K, Nishimoto Y, Uzura A, Kaneko S (2009). A beta-l-arabinopyranosidase from *Streptomyces avermitilis* is a novel member of glycoside hydrolase family 27. J Biol Chem.

[CR32] Jiang D, Fan J, Wang X, Zhao Y, Huang B, Liu J, Zhang XC (2012a) Crystal structure of 1,3Gal43A, an exo-β-1,3-galactanase from *Clostridium thermocellum*. J Struct Biol 180:447-457 10.1016/j.jsb.2012.08.005 22960181

[CR33] Jiang SS, Liang X N, Li X, Wang S L, Lv D W, Ma CY, Li XH, MaWJ, Yan YM (2012b) Wheat drought-responsive grain proteome analysis by linear and nonlinear 2-DE and MALDI-TOF mass spectrometry. Int J Mol Sci 13:16065-16083 10.3390/ijms131216065 PMC3546679 23443111

[CR34] Jain M, Nijhawan A, Arora R, Agarwal P, Ray S, Sharma P, Kapoor S, Tyagi AK, Khurana JP (2007) F-box proteins in rice. Genome-wide analysis, classification, temporal and spatial gene expression during panicle and seed development, and regulation by light and abiotic stress. Plant Physiol 143:1467-1483 10.1104/pp.106.091900 PMC1851844 17293439

[CR35] Jung KH, Gho HJ, Giong HK, Chandran AK, Nguyen QN, Choi H, Zhang T, Wang W, Kim JH, Choi HK, An G (2013). Genome-wide identification and analysis of Japonica and Indica cultivar-preferred transcripts in rice using 983 Affymetrix array data. Rice.

[CR36] Kawahara Y, de la Bastide M, Hamilton JP, Kanamori H, McCombie WR, Ouyang S, Schwartz DC, Tanaka T, Wu J, Zhou S, Childs KL, Davidson RM, Lin H, Quesada-Ocampo L, Vaillancourt B, Sakai H, Lee SS, Kim J, Numa H, Itoh T, Buell CR, Matsumoto T (2013). Improvement of the *Oryza sativa* Nipponbare reference genome using next generation sequence and optical map data. Rice.

[CR37] Kawahara Y, Oono Y, Wakimoto H, Ogata J, Kanamori H, Sasaki H, Mori S, Matsumoto T, Itoh T (2016). TENOR: database for comprehensive mRNA-Seq experiments in rice. Plant Cell Physiol.

[CR38] Krieger E, Koraimann G, Vriend G (2002). Increasing the precision of comparative models with YASARA NOVA - a self-parameterizing force field. Proteins.

[CR39] Krogh A, Larsson B, von Heijne G, Sonnhammer EL (2001). Predicting transmembrane protein topology with a hidden Markov model: application to complete genomes. J Mol Biol.

[CR40] Kyndt T, Denil S, Haegeman A, Trooskens G, Bauters L, Van Criekinge W, De Meyer T, Gheysen G (2012). Transcriptional reprogramming by root knot and migratory nematode infection in rice. New Phytol.

[CR41] Lannoo N, Van Damme EJM (2010). Nucleocytoplasmic plant lectins. Biochim Biophys Acta.

[CR42] Lannoo N, Van Damme EJM (2014). Lectin domains at the frontiers of plant defense. Front Plant Sci.

[CR43] Laskowski RA, MacArthur MW, Moss DS, Thornton JM (1993). PROCHECK: a program to check the stereochemistry of protein structures. J Appl Cryst.

[CR44] Lee K, Lam KH, Kruel AM, Perry K, Rummel A, Jin R (2014). High-resolution crystal structure of HA33 of botulinum neurotoxin type B progenitor toxin complex. Biochem Biophys Res Commun.

[CR45] Lee TH, Tang H, Wang X, Paterson AH (2013). PGDD: a database of gene and genome duplication in plants. Nucleic Acids Res.

[CR46] Li D, Wang X, Yuan D, Zhang L, Jiang X, Tao Z, Li Y, Wang J, Li X, Yang Y (2014). Over-expression of ArathEULS3 confers ABA sensitivity and drought tolerance in Arabidopsis. Plant Cell Tiss Org.

[CR47] Liu F, Xu W, Wei Q, Zhang Z, Xing Z, Tan L, Di C, Yao D, Wang C, Tan Y, Lin Y, Sun C, Xue Y, Su Z (2010). Gene expression profiles deciphering rice phenotypic variation between Nipponbare (Japonica) and 93-11 (Indica) during oxidative stress. PLoS ONE.

[CR48] Liu C, Mao B, Ou S, Wang W, Liu L, Wu Y, Chu C, Wang X (2014). OsbZIP71, a bZIP transcription factor, confers salinity and drought tolerance in rice. Plant Mol Biol.

[CR49] Lu G, Gao C, Zheng X, Han B (2009). Identification of OsbZIP72 as a positive regulator of ABA response and drought tolerance in rice. Planta.

[CR50] Marcel S, Sawers R, Oakeley E, Angliker H, Paszkwoski U (2010). Tissue-adapted invasion strategies of the rice blast fungus *Magnaporthe oryzae*. Plant Cell.

[CR51] Mathelier A, Fornes O, Arenillas DJ, Chen CY, Denay G, Lee J, Shi W, Shyr C, Tan G, Worsley-Hunt R, Zhang AW, Parcy F, Lenhard B, Sandelin A, Wasserman WW (2015). JASPAR 2016: a major expansion and update of the open-access database of transcription factor binding profiles. Nucl Acid Res.

[CR52] Matys V, Fricke E, Geffers R, Gössling E, Haubrock M, Hehl R, Hornischer K, Karas D, Kel AE, Kel-Margoulis OV, Kloos DU, Land S, Lewicki-Potapov B, Michael H, Münch R, Reuter I, Rotert S, Saxel H, Scheer M, Thiele S, Wingender E (2003). TRANSFAC®: transcriptional regulation, from patterns to profiles. Nucl Acid Res.

[CR53] Melo F, Feytmans E (1998). Assessing protein structures with a non-local atomic interaction energy. J Mol Biol.

[CR54] Mitchell AL, Chang HY, Daugherty L, Fraser M, Hinter S, Lopez R, McAnulla C, McMenamin C, Nuka G, Pesseat S, Sangrador-Vegas A, Scheremetjew M, Rato C, Yong SY, Bateman A, Punta M, Attwood TK, Sigrist CJ, Redaschi N, Rivoire C, Xenarios I, Kahn D, Guyot D, Bork P, Letunic I, Gough J, Oates M, Haft D, Huang H, Natale DA, Wu CH, Orengo C, Sillitoe I, Mi H, Thomas PD, Finn RD (2015). The InterPro protein families database: The classification resource after 15 years. Nuc Acid Res.

[CR55] Molina C, Grotewold E (2005). Genome wide analysis of Arabidopsis core promoters. BMC Genomics.

[CR56] Moons A, Gielen J, Vandekerckhove J, Van Der Straeten D, Gheysen G, Van Montagu M (1997). An abscisic-acid-and salt-stress-responsive rice cDNA from a novel plant gene family. Planta.

[CR57] Nakamura T, Tonozuka T, Ito S, Takeda Y, Sato R, Matsuo I, Ito Y, Oguma K, Nishikawa A (2011). Molecular diversity of the two sugar-binding sites of the β-trefoil lectin HA33/C (HA1) from *Clostridium botulinum* type C neurotoxin. Arch Biochem Biophys.

[CR58] Nijhawan A, Jain M, Tyagi AK, Khurana JP (2008). Genomic survey and gene expression analysis of the basic leucine zipper transcription factor family in rice. Plant Physiol.

[CR59] Palaniswamy SK, James S, Sun H, Lamb RS, Davuluri RV, Grotewold E (2006). AGRIS and AtRegNet. a platform to link cis-regulatory elements and transcription factors into regulatory networks. Plant Physiol.

[CR60] Petersen TN, Brunak S, von Heijne G, Nielsen H (2011). SignalP 4.0: discriminating signal peptides from transmembrane regions. Nat Methods.

[CR61] Petryniak J, Pereira ME, Kabat EA (1977). The lectin of *Euonymus europeus*: purification, characterization, and an immunochemical study of its combining site. Arch Biochem Biophys.

[CR62] Pettersen EF, Goddard TD, Huang CC, Couch GS, Greenblatt DM, Meng EC, Ferrin TE (2004). UCSF Chimera - a visualization system for exploratory research and analysis. J Comput Chem.

[CR63] Proost S, Van Bel M, Vaneechoutte D, Van de Peer Y, Inzé D, Mueller-Roeber B, Vandepoele K (2015). PLAZA 3.0: an access point for plant comparative genomics. Nucl Acid Res.

[CR64] Quinlan AR, Hall IM (2010). BEDTools: a flexible suite of utilities for comparing genomic features. Bioinformatics.

[CR65] Rabello AR, Guimarães CM, Rangel PH, da Silva FR, Seixas D, de Souza E, Brasileiro AC, Spehar CR, Ferreira ME, Mehta A (2008). Identification of drought-responsive genes in roots of upland rice (*Oryza sativa* L.). BMC Genomics.

[CR66] Ray S, Dansana PK, Giri J, Deveshwar P, Arora R, Agarwal P, Khurana JP, Kapoor S, Tyagi AK (2011). Modulation of transcription factor and metabolic pathway genes in response to water-deficit stress in rice. Funct Integr Genomics.

[CR67] RoyChoudhury A, Gupta B, Sengupta DN (2008). Trans-acting factor designated OSBZ8 interacts with both typical abscisic acid responsive elements as well as abscisic acid responsive element-like sequences in the vegetative tissues of indica rice cultivars. Plant Cell Rep.

[CR68] Sagane Y, Hayashi S, Akiyama T, Matsumoto T, Hasegawa K, Yamano A, Suzuki T, Niwa K, Watanabe T, Yajima S (2016). Conformational divergence in the HA-33/HA-17 trimer of serotype C and D botulinum toxin complex. Biochem Biophys Res Commun.

[CR69] Steffens NO, Galuschka C, Schindler M, Bülow L, Hehl R (2004). AthaMap: an online resource for in silico transcription factor binding sites in the *Arabidopsis thaliana* genome. Nucl Acid Res.

[CR70] Stolzer M, Lai H, Xu M, Sathaye D, Vernot B, Durand D (2012). Inferring duplications, losses, transfers, and incomplete lineage sorting with non-binary species trees. Bioinformatics.

[CR71] Suzuki R, Fujimoto Z, Ito S, Kawahara S, Kaneko S, Taira K, Hasegawa T, Kuno A (2009). Crystallographic snapshots of an entire reaction cycle for a retaining xylanase from *Streptomyces olivaceoviridis* E-86. J Biochem.

[CR72] Treiber N, Reinert DJ, Carpusca I, Aktories K, Schulz GE (2008). Structure and mode of action of a mosquitocidal holotoxin. J Mol Biol.

[CR73] Van Damme EJM, Lannoo N, Peumans WJ (2008). Plant lectins. Adv Bot Res.

[CR74] Van de Velde J, Heyndrickx KS, Vandepoele K (2014). Interference of transcriptional networks in Arabidopsis through conserved noncoding sequence analysis. Plant Cell.

[CR75] Van Holle S, Van Damme EJM (2015). Distribution and evolution of the lectin family in soybean (*Glycine max*). Molecules.

[CR76] Van Hove J, Fouquaert E, Smith DF, Proost P, Van Damme EJM (2011). Lectin activity of the nucleocytoplasmic EUL protein from *Arabidopsis thaliana*. Biochem Biophys Res Commun.

[CR77] Van Hove J, Stefanowicz K, De Schutter K, Eggermont L, Lannoo N, Al Atalah B, Van Damme EJM (2014). Transcriptional profiling of the lectin ArathEULS3 from *Arabidopsis thaliana* toward abiotic stresses. J Plant Physiol.

[CR78] Van Hove J, De Jaeger G, De Winne N, Guisez Y, Van Damme EJM (2015). The Arabidopsis lectin EULS3 is involved in stomatal closure. Plant Sci.

[CR79] Vandepoele K, Quimbaya M, Casneuf T, De Veylder L, Van de Peer Y (2009). Unraveling transcriptional control in Arabidopsis using cis-regulatory elements and coexpression networks. Plant Physiol.

[CR80] Vítámvás P, Urban MO, Škodáček Z, Kosová K, Pitelková I, Vítámvás J, Renaut J, Prášil IT (2015). Quantitative analysis of proteome extracted from barley crowns grown under different drought conditions. Front Plant Sci.

[CR81] Voorrips RE (2002). MapChart: Software for the Graphical Presentation of Linkage Maps and QTLs. J Hered.

[CR82] Weirauch MT, Yang A, Albu M, Cote AG, Montenegro-Montero A, Drewe P, Najafabadi HS, Lambert SA, Mann I, Cook K, Zheng H, Goity A, van Bakel H, Lozano JC, Galli M, Lewsey MG, Huang E, Mukherjee T, Chen X, Reece-Hoyes JS, Govindarajan S, Shaulsky G, Walhout AJ, Bouget FY, Ratsch G, Larrondo LF, Ecker JR, Hughes TR (2014). Determination and inference of eukaryotic transcription factor sequence specificity. Cell.

[CR83] Yang Y, Zhu K, Xia H, Chen L, Chen K (2014). Comparative proteomic analysis of indica and japonica rice varieties. Genet Mol Biol.

[CR84] Yazaki J, Kikuchi S, Litwack G (2005). The genomic view of genes responsive to the antagonistic phytohormones, abscicic acid, and gibberellin. Plant Hormones.

[CR85] Zhang W, Wu Y, Schnable JC, Zeng Z, Freeling M, Crawford GE, Jiang J (2012). High-resolution mapping of open chromatin in the rice genome. Genome Res.

[CR86] Zhou YL, Xu MR, Zhao MF, Xie XW, Zhu LH, Fu BY, Li ZK (2010). Genome-wide gene responses in a transgenic rice line carrying the maize resistance gene Rxo1 to the rice bacterial streak pathogen, Xanthomonas oryzae pv. oryzicola. BMC Genomics.

