# Supplementary figures and images for: Evolutionary relationships and expression analysis of EUL domain proteins in rice (Oryza sativa)
Source: Rice (N Y). 2017 May 30;10:26. doi: 10.1186/s12284-017-0164-3 (PMC5449364; doi:10.1186/s12284-017-0164-3)

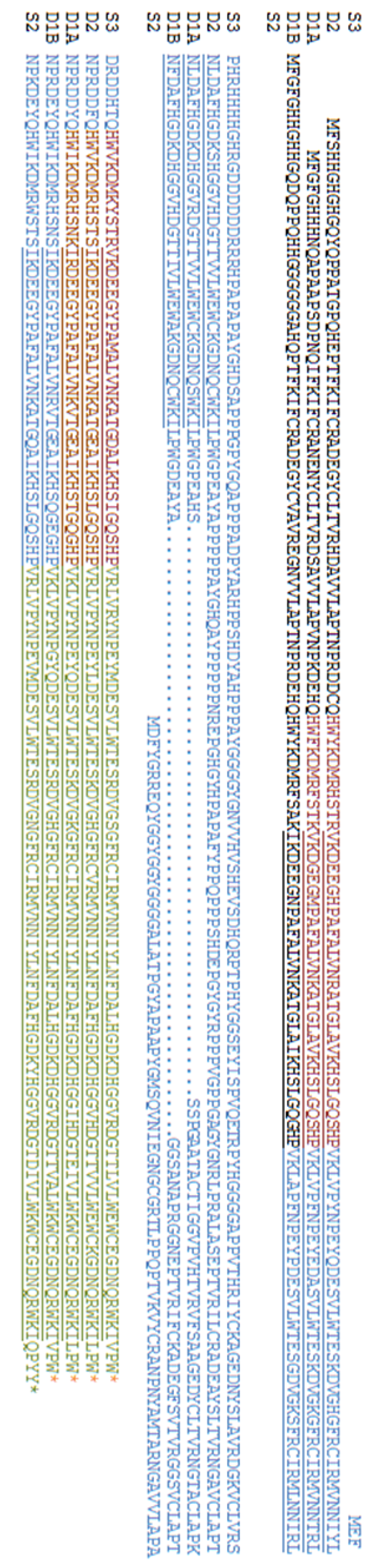

Supplement: Supplementary file 2 — Exon structure plotted on the protein sequences of the rice EULs. Sequences encoded by different exons are shown in different colors, the sequence corresponding to the EUL domain (pFam) is underlined. (PNG 1719 kb) [file 12284_2017_164_MOESM2_ESM.png]

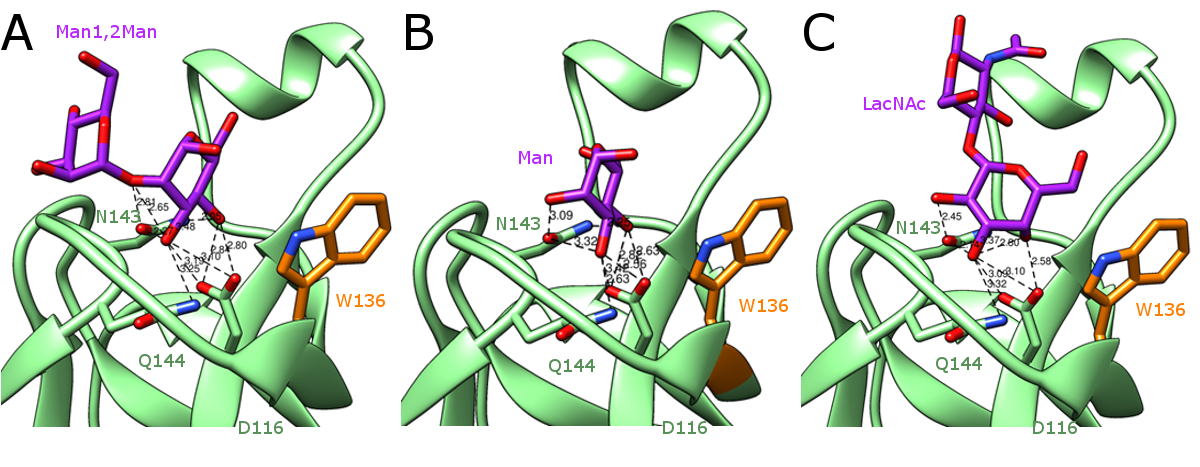

Supplement: Supplementary file 5 — Binding models of selected ligands in the carbohydrate-binding site of OsEULS3. A-C. Side-view of the carbohydrate-binding site of OsEULS3 showing the docking of Man1,2Man (A), Man (B) or LacNAc (C) to the carbohydrate-binding site. The H-bond distances are indicated (Å). A stacking interaction occurs between the first Man ring (A), Man (B) or Gal (C) and the aromatic residues F118 and W136 (colored orange) located in the vicinity of the active site. (PNG 457 kb) [file 12284_2017_164_MOESM5_ESM.png]

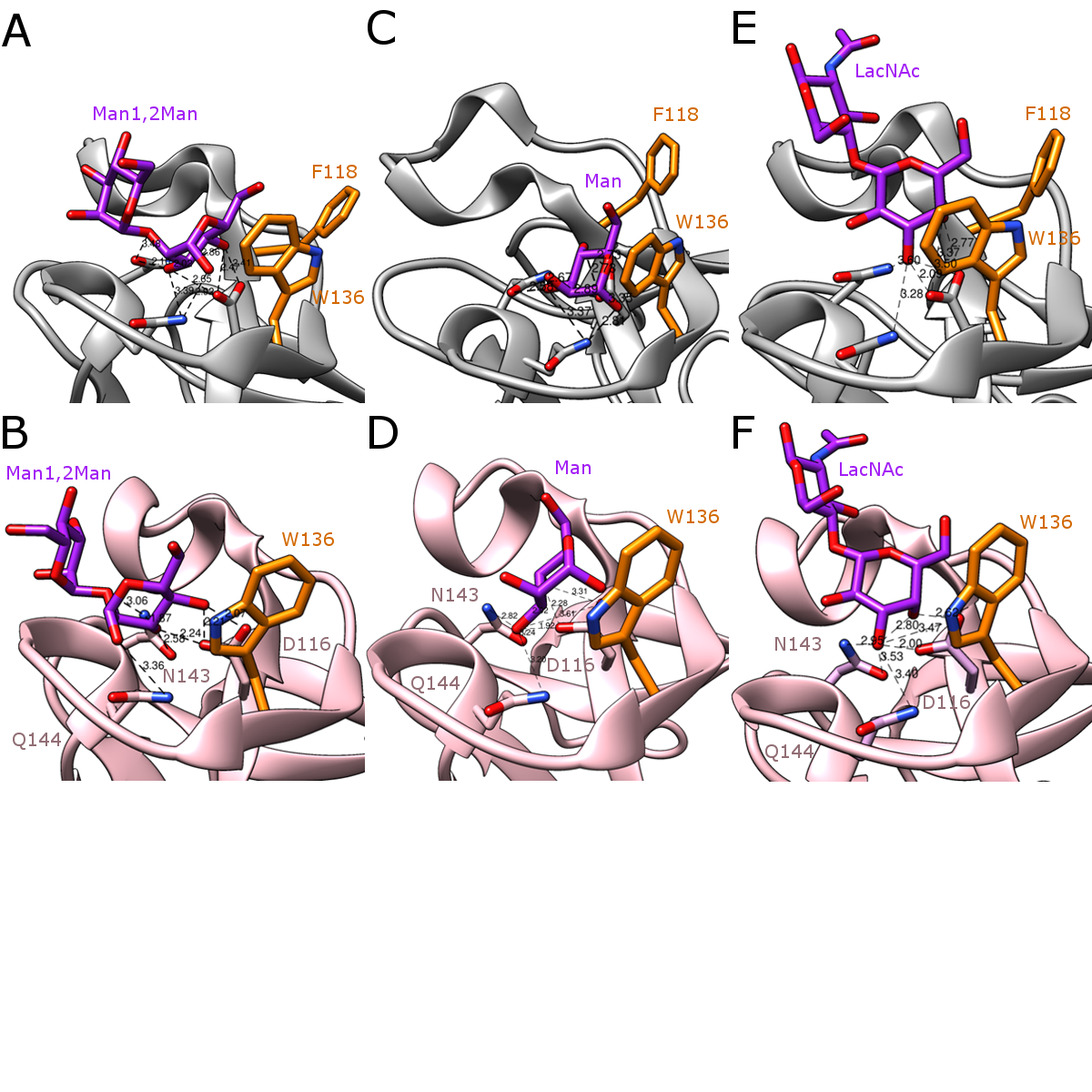

Supplement: Supplementary file 6 — Binding models of selected ligands in the carbohydrate-binding site of OsEULD2. A, C and E. Side-view of the carbohydrate-binding site of OsEULD2_1 showing the docking of Man1,2Man (A), Man (C) or LacNAc (E) to the carbohydrate-binding site. A stacking interaction occurs between the first Man ring (A), Man (C) or Gal (E) and the aromatic residues F118 and W136 (colored orange) located in the vicinity of the active site. B, D and F. Side-view of the carbohydrate-binding site of OsEULD2_2 showing the docking of Man1,2Man (B), Man (D) or LacNAc (F) to the carbohydrate-binding site. A stacking interaction occurs between the first Man ring (B), Man (D) or Gal (F) and W136 (colored orange) located in the vicinity of the active site. The H-bond distances are indicated (Å). (PNG 853 kb) [file 12284_2017_164_MOESM6_ESM.png]

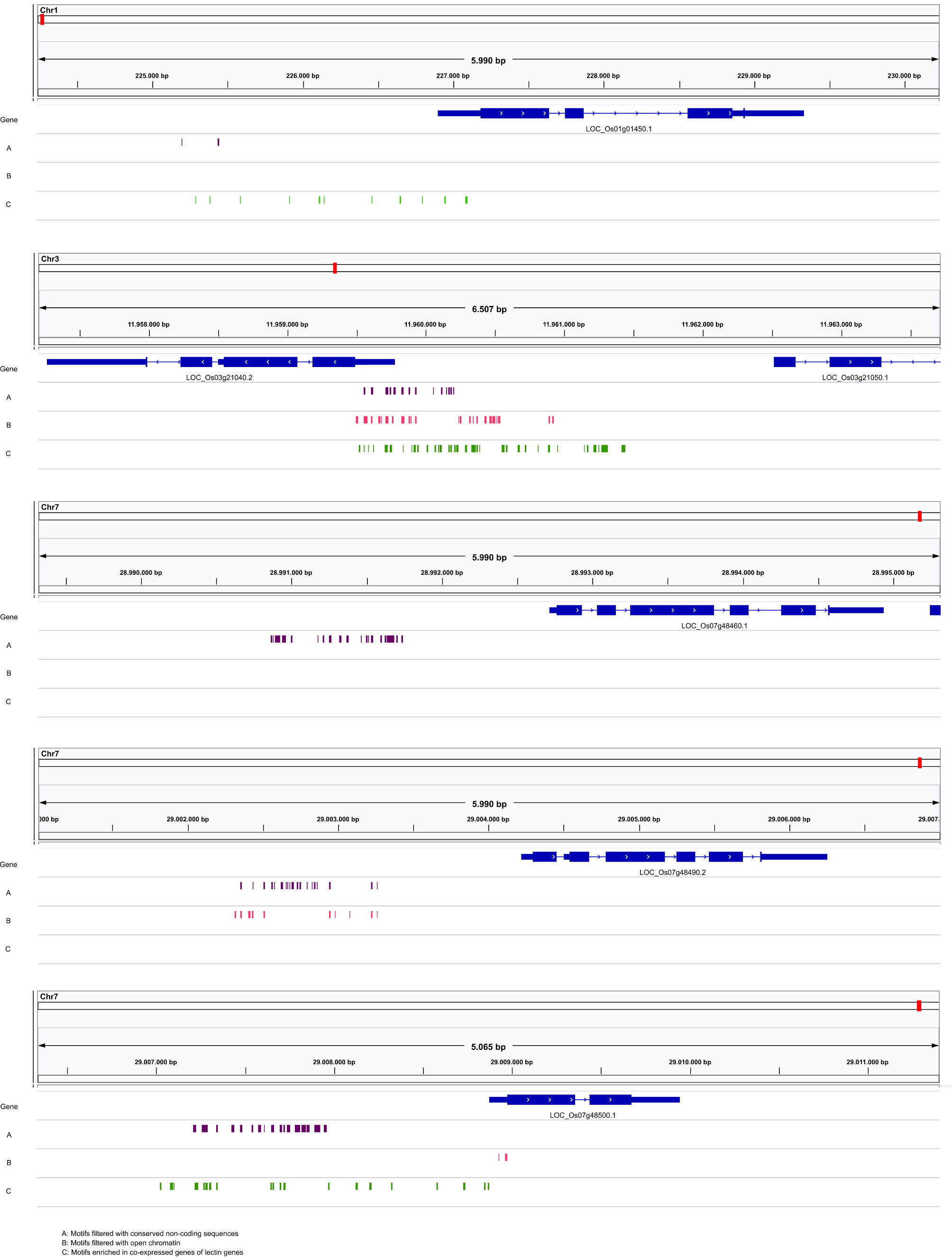

Supplement: Supplementary file 8 — Overview of different cis-regulatory elements mapped on the rice lectin genes. Elements identified in the promoters by the different analyses performed in the integrated approach: motifs filtered with conserved non-coding sequences (track A), motifs filtered with open chromatin (track B) and motifs enriched in co-expressed genes of lectin genes (track C). (PNG 191 kb) [file 12284_2017_164_MOESM8_ESM.png]

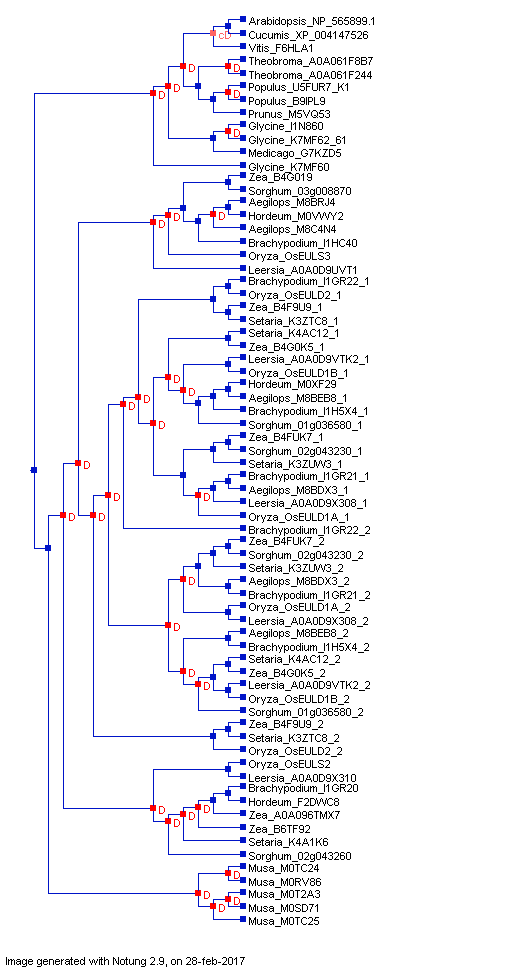

Supplement: Supplementary file 12 — Reconciliated tree. Phylogenetic tree of the EUL domains reconciliated with the species tree using Notung 2.9 (Stolzer et al. 2012). Duplication events are indicated with “D”. (PNG 19 kb) [file 12284_2017_164_MOESM12_ESM.png]
